# Privacy-preserving biological age prediction over federated human methylation data using fully homomorphic encryption

**DOI:** 10.1101/gr.279071.124

**Published:** 2024-09

**Authors:** Meir Goldenberg, Loay Mualem, Amit Shahar, Sagi Snir, Adi Akavia

**Affiliations:** 1 Department of Computer Science, The University of Haifa, Haifa 3103301, Israel;; 2 Department of Evolutionary and Environmental Biology, The University of Haifa, Haifa 3103301, Israel

## Abstract

DNA methylation data play a crucial role in estimating chronological age in mammals, offering real-time insights into an individual's aging process. The epigenetic pacemaker (EPM) model allows inference of the biological age as deviations from the population trend. Given the sensitivity of this data, it is essential to safeguard both inputs and outputs of the EPM model. A privacy-preserving approach for EPM computation utilizing fully homomorphic encryption was recently introduced. However, this method has limitations, including having high communication complexity and being impractical for large data sets. The current work presents a new privacy-preserving protocol for EPM computation, analytically improving both privacy and complexity. Notably, we employ a single server for the secure computation phase while ensuring privacy even in the event of server corruption (compared to requiring two noncolluding servers in prior work). Using techniques from symbolic algebra and number theory, the new protocol eliminates the need for communication during secure computation, significantly improves asymptotic runtime, and offers better compatibility to parallel computing for further time complexity reduction. We implemented our protocol, demonstrating its ability to produce results similar to the standard (insecure) EPM model with substantial performance improvement compared to prior work. These findings hold promise for enhancing data security in medical applications where personal privacy is paramount. The generality of both the new approach and the EPM suggests that this protocol may be useful in other applications employing similar expectation–maximization techniques.

Genomic data are protected by privacy regulations, such as the European General Data Protection Regulation (GDPR) (https://eur-lex.europa.eu/eli/reg/2016/679/oj), the California Consumer Privacy Act (CCPA) (https://oag.ca.gov/privacy/ccpa), and the Genetic Information Privacy Act (GIPA) (https://www.congress.gov/bill/116th-congress/house-bill/2155), that may limit the ability of companies and researchers to collect large cohorts of genomic data as required for training machine learning models of high predictive power. A promising approach for overcoming such limitations is to execute *privacy-preserving genome analysis*, that is, to utilize cryptographic techniques such as fully homomorphic encryption (FHE) (Rivest et al. 1978; Gentry 2009) that enable processing data in encrypted form, so that even the entity processing it is never exposed to the underlying data in cleartext. Prior work on privacy-preserving genome analysis using homomorphic encryption focused primarily on genome-wide association studies (GWAS) (Lu et al. 2015; Simmons and Berger 2016; Bonte et al. 2018; Blatt et al. 2020; Dong et al. 2022)—that is, statistically associating innate genome variability in single-nucleotide polymorphisms (SNPs) with a risk for a disease or a particular trait—as well as on privacy-preserving classification of DNA and RNA sequences of tumor tissues (Carpov et al. 2022; Hong et al. 2022) and viral strains (Zhou et al. 2022; Akavia et al. 2023), respectively. Recently, privacy-preserving epigenetics—that is, the study of how behavior and environmental factors lead to genome changes that in turn affect the phenotype—was considered in Goldenberg et al. (2022) and Akavia et al. (2024), analyzing gene expression data (Akavia et al. 2024) and DNA methylation data (Goldenberg et al. 2022).

Elaborating on the latter, DNA methylation is a chemical change in the genome that is linked to numerous developmental, physiologic, and pathologic processes, including malignancy, infections, and aging. A breakthrough in this area was achieved by the *epigenetic clock* of Horvath (2013) that predicted human chronological age based on observing several hundreds of methylation sites along the genome. Seeking to link between these methylation processes and aging, Snir et al. (2016) suggested a flexible, probabilistic framework adopted from the evolutionary realm (Snir et al. 2012, 2014; Wolf et al. 2013), under which a maximum likelihood solution is sought, called the epigenetic pacemaker (EPM). The output of the algorithm is a point in the likelihood surface under which the probability of the entire system is optimized while preserving basic model constraints. The flexibility of the framework enabled explaining intrinsic key features in aging, both in human and animals (Snir et al. 2019; Pinho et al. 2022). In Snir and Pellegrini (2018), an expectation–maximization (EM) algorithm was proposed to search the likelihood surface via several iterations consisting of two steps, each increasing the likelihood function. Subsequently, using algebraic identities, along with the special structure of the EPM framework, closed-form solutions to both steps were found, yielding an asymptotic reduction in time and space complexities (Snir 2020). One of the outputs of the EPM framework is the *epigenetic state* (also referred to as the “biological age,” to be contrasted with the chronological one). The epigenetic state can provide significant information on the health status of an individual, which can be utilized, for example, in estimating the efficacy of pharmaceutical interventions or in predicting life expectancy. Accurate estimations and predictions may require, however, a larger data set than available for a single entity such as a hospital or a research laboratory, making it desirable to combine data sets from several entities, albeit, without compromising privacy. Concretely, our experiments on synthetic data show that thousands of individuals are required for achieving good accuracy in EPM prediction; details appear in Supplemental Note 5. To address the privacy issue a first step was made in Goldenberg et al. (2022), proposing a privacy-preserving protocol for computing the EPM model using FHE as a central tool. Their protocol required two servers throughout the entire computation, resulting in two main drawbacks: first, their protocol is insecure if an adversary corrupts both servers; second, their protocol suffers from a high communication and computational toll, making it impractical to real data volumes.

In this work, we propose a new privacy-preserving protocol for the EPM, improving over the prior work (Goldenberg et al. 2022) in the following three aspects:

1. *Improved complexity: no communication*. In Goldenberg et al. (2022), secure computing entails multiple rounds of communication between the two servers: one communication round for each EM iteration in computing the EPM, transmitting *O*(*nm*) ciphertexts in each iteration (for *n* the number of methylation sites, and *m* the number of individuals in the data set). In contrast, our secure computing entails *no communication* between the two servers, regardless of the number of EM iterations securely computed (cf. “Privacy-preserving EPM: protocol”).
2. *Stronger privacy: hiding both input and output*. In Goldenberg et al. (2022), the produced epigenetic ages (e-ages) are revealed to all parties. In contrast, we offer an enhanced security version of our protocol where we *protect the privacy of both input and output*. That is, rather than revealing all e-ages to all parties, we reveal each e-age only to the entity who provided the data for the corresponding individual. This strengthening requires only a single round of interaction between the servers, transmitting a single ciphertext in each direction. Moreover, if we slightly relax the output hiding requirement as to allow exposing the common denominator of the produced e-ages, then this privacy enhancement incurs no interaction.
3. *Stronger privacy: relying on a more plausible assumption*. Both ours and Goldenberg et al. (2022)’s security guarantee relies on an assumption that the two servers are noncolluding; that is, an adversary can corrupt one server or the other, but not both. In Goldenberg et al. (2022), both servers are required to be online and actively participate throughout the entire protocol, including during the computing phase which entails the bulk of the computation; this often makes a noncollusion assumption challenging to realize. In contrast, in our protocol only one server executes the secure computing phase, while the other server's role is essentially minimized to be the role of a key management service (KMS), that is, key generation and decryption in the pre- and postprocessing phases, respectively, whereas it can be offline and idle throughout the secure computing phase. Being offline throughout the bulk of the computation makes it considerably easier to safeguard this server, thus increasing the plausibility of the noncollusion assumption. In further detail, we present three variants of our protocol. In two of those variants, the role of the second server is exactly that of a KMS. In the third variant, this second server executes one additional computation: computing the modular inverse of a single (masked) output value.

We implemented our protocol and show that it offers a substantial performance speedup over the implementation of Goldenberg et al. (2022): securely computing the EPM from 716 methylation sites in 3 hours (cf. 24 sites in 3 hours in Goldenberg et al. [2022]). These results can serve as a pilot for data security measures integrated in vast medical applications where personal privacy is imperative.

In terms of techniques, we focus on offering a new algorithm for computing the EPM model, which avoids the complexity bottlenecks of the underlying FHE (such as computing matrix inverse and division). Concretely, our algorithm substitutes matrix inverse computations with closed-form algebraic formulas that were computed analytically building on Snir (2020), and substitutes division computations by representing rational numbers as pairs of integers—their numerator and denominator. We note that the above interventions lead to high magnitude numbers throughout the computation, which we reduce to a manageable scale using the Chinese Remainder Theorem (CRT).

## Methods

This work integrates the EPM with FHE. In this section, we will discuss and define these methodologies and present the problem definition.

### The epigenetic pacemaker

We summarize the EPM model and optimization problem (Snir et al. 2016). Let *s*_1_, …, *s*_*n*_ be *methylation sites* in the genome that exist in every individual and undergo methylation during life at characteristic *rate r*_*i*_. Each site *s*_*i*_ is initiated at birth with an *initial level* of methylation, denoted $s_{i}^{0}$. Both $s_{i}^{0}$ and *r*_*i*_ are universal—common to all individuals. The actual rate for a specific individual may vary. However, the *EPM property* mandates that site rates change proportionally throughout the lifetime over all sites of the same individual. That is, at any point in time, if a change in rate occurred in site *i* of individual *j*, then the rates in *all* sites $i^{\prime }$ in *j* are simultaneously changed and by the same factor, so that the ratio between any two rates *r*_*i*_ and $r_{i^{\prime }}$ is maintained at all times. Although methylation rates might even be negative (demethylation), it is these changes in the rates that are correlated with the *aging rate*, providing a good estimate on the *e-age* of an individual (as opposed to its chronological age) (Snir et al. 2016). We denote by *t*_*j*_ the weighted average e-age of individual *j*, accounting for the rate changes an individual has undergone through life.

The algorithmic task under the EPM model is to find the maximum likelihood values of $s_{i}^{0}$, *r*_*i*_, and *t*_*j*_, when given the observed methylation levels in *n* genome sites as measured in *m* individuals. The input is denoted by ${\left({\hat{s}}_{i,j}\right)}_{\;j\in [m],i\in [n]}$, where ${\hat{s}}_{i,j}$ denotes the methylation measured in individual *j* at site *i* (where [*x*] = 1, 2, 3, …, *x*).

An algorithm for the EPM optimization problem was presented by Snir et al. (2016), who show that their algorithm provably converges to a local optima of the maximum likelihood function. Subsequently, Snir (2020) presented an experimental evaluation validating the concrete good efficiency of this algorithm. This algorithm is the starting point of our solution.

To describe the algorithm, we first organize the observed variables ${\hat{s}}_{ij}$ as well as the unknown variables *t*_*j*_, *r*_*i*_, and $s_{i}^{0}$ as follows. Let *X* be a *mn* × 2*n* matrix whose *k*th row is all zero except for the value *t*_*j*_ in the *i*th entry of its first half and 1 in the *i*th entry of its second half. Let β be a column vector whose first *n* entries are *r*_1_, …, *r*_*n*_ and the last *n* entries are $s_{1}^{0},\ldots ,s_{n}^{0}$. Let *y* be the column vector whose im+j entry contains *s*_*i*,*j*_. See Supplemental Figure S1.

The algorithm of Snir (2020) consists of several iterations, where in each iteration the algorithm alternates between two main components: a *site step* and a *time step*. In the site step, values for *t*_*j*_’s are fixed to be the values obtained from the previous iteration (on the first iteration, they are initialized to random values), and the algorithm solves the linear-regression problem system specified by *X*, *y* (where *X* is with the said values for *t*_*j*_’s) to obtain values from *r*_*i*_ and $s_{i}^{0}$ (i.e., for β). In the time step, *r*_*i*_ and $s_{i}^{0}$ are fixed to the values obtained in the site step (of the current iteration), and the individual's times are set to their maximum likelihood values, which as proved by Snir (2020), are given by the following closed-form rational function:

(1) $$t_{j}=\frac{\sum_{i=1}^{n}r_{i}({\hat{s}}_{i,j}-s_{i}^{0})}{\sum_{i=1}^{n}r_{i}^{2}}.$$

Furthermore, Snir (2020) proves that at every such step an increase in the likelihood is guaranteed, and so, a local optimum is eventually reached. The iterations can proceed until the improvement in the residual sum of square (RSS) falls below a threshold δ given as a parameter to the algorithm.

Our goal is to compute the e-age in a *privacy-preserving* fashion. Therefore, we must not reveal even the number of iterations required for convergence, because this could potentially reveal significant information on the input (see Akavia et al. [2024] for discussion of such attacks). We, therefore, slightly modify the algorithm from Snir (2020), in specifying the number of iterations in advance, by a user-defined parameter denoted iter. This cleartext algorithm is summarized in Supplemental Figure S2.

### Privacy-preserving EPM: definition

We summarize the privacy-preserving EPM over federated data problem introduced in Goldenberg et al. (2022).

#### Privacy-preserving EPM: settings and goal

**Figure 1.**
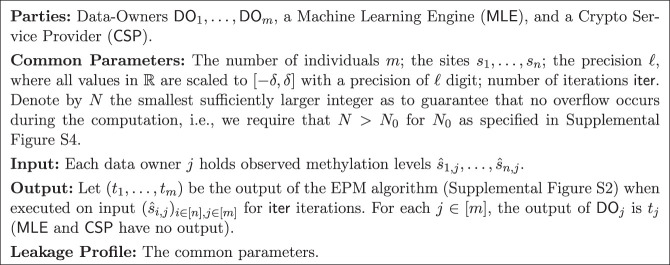
EPM functionality.

There are *m* individuals, called *Data Owners*, denoted by DO_1_, …, DO_*m*_. Each data owner DO_*j*_ holds observed methylation levels ${\hat{s}}_{1,j},\ldots ,\;{\hat{s}}_{n,j}$ in *n* sites *s*_1_, …, *s*_*n*_ (all measurements are for a known and identical set of genome sites). The data owners wish to compute the e-age estimator specified by the EPM algorithm on their joint data, but without revealing information on their individual data. The data owners encrypt the data and outsource the computation to a server called the machine learning engine (MLE) that executes the computation, whereas the complexity of the data owners is proportional only to the size of their individual input (in encrypted form). The parties also have access to a crypto service provider (CSP) that can generate key pairs and decrypt for authorized values (alternatively, provide decryption keys to authorized parties). The goal is to compute the same e-age estimation as outputted by the EPM algorithm when executed on the union of the individual data, but without exposing any information on the raw data (beyond what can be inferred from the designated output and leakage profile). This is summarized in the *EPM functionality* depicted in Figure 1.

#### Threat model and security requirement

To achieve the above goal, the parties engage in an interactive protocol in which parties can repeatedly send messages to each other and execute local computations on their input and received messages. We analyze security in the two-server model, as in Goldenberg et al. (2022); that is, the security requirement is to guarantee correctness and privacy against all passive computationally bounded adversaries who may corrupt any subset of the data owners and at most one out of the CSP and MLE. Being passive means that parties controlled by the adversary follow the protocol specification, albeit they may collude to infer as much information as possible from their view of the interaction. Being computationally bounded means that all parties are restricted to performing probabilistic polynomial time computations.

To capture this formally we first specify some standard terminology. Let Π be a protocol for computing EPM; denote by *x*_1_, …, *x*_*m*_ the inputs of DO_1_, …, DO_*m*_; and λ the security parameter. The output in an execution of Π on these inputs and security parameter is a random variable denoted by $\mathrm{outpu}t^{\Pi }(x_{1},\ldots ,x_{m})$ (where the probability here is over the randomness of all participating parties, including the servers). The output of the EPM functionality (cf. Fig. 1) on these inputs is a random variable denoted by EPM(*x*_1_, …, *x*_*m*_) (where the probability is over the randomness of the EPM algorithm (cf. Supplemental Fig. S2), and we denote by EPM(*x*_1_, …, *x*_*m*_)_*j*_ its *j*th coordinate which is output of DO_*j*_. The *correctness* requirement is that with overwhelming probability the output of the protocol is identical to the output of EPM (see Definition 1, Correctness). The *view* of any party *P* ∈ {DO_1_, …, DO_*m*_, MLE, CSP} during an execution of Π on inputs *x*_1_, …, *x*_*m*_ of DO_1_, …, DO_*m*_, respectively, and security parameter λ is the random variable consisting of the *input* and *randomness* of *P* and the *messages P received* from the other parties during the execution of Π, and denoted by ${\mathrm{view}}_{C}^{\Pi }(x_{1},\ldots ,x_{m}).$ The *privacy* requirement is that for any set C of corrupt parties that includes at most one of the two servers, the view of C is computationally indistinguishable from a random variable that can be efficiently computed when given only the input and output of corrupt parties and the leakage L (as specified in Fig. 1). This captures the property that participating in the protocol does not equip the corrupt parties with any further knowledge. See Definition 1, Privacy.

Definition 1 **(Securely realizing EPM).**

*We say that a protocol* Π securely realizes the *EPM* functionality (*cf.* Fig. 1) *with leakage* L *against passive computationally bounded adversaries in the two-server model if the following holds:*

1. *Correctness: There exists a negligible function* negl (λ): ℕ → ℕ *such that for all inputs* $\vec{x}=(x_{1},\ldots ,x_{m})$ *and security parameter* λ,
  $$\mathrm{Pr}[\mathrm{outpu}t^{\Pi }(\vec{x})=\mathrm{EPM}\;(\vec{x})]=1-negl\;(\lambda )$$
  *(where the probability is over the randomness of the parties in the protocol and over the randomness of the EPM algorithm).*
2. *Privacy: For every set of corrupt parties* $C\subset \{DO_{1},\ldots ,$ $DO_{m},MLE,CSP\}$ *consisting of any number of data owners and at most one of MLE and CSP, there exists a computationally bounded simulator Sim such that for every input* $\vec{x}=(x_{1},\ldots ,x_{m})$,
  $$\begin{array}{rl} & ({\mathrm{view}}_{C}^{\Pi }(\vec{x}),\mathrm{outpu}t^{\Pi }(\vec{x}))\approx_{c}\\ & (\mathrm{Sim}({\left(x_{j},\mathrm{EPM}{\left(\vec{x}\right)}_{j}\right)}_{DO_{j}\in C},L),\mathrm{EPM}(\vec{x})), \end{array}$$
  *where* ≈_*c*_ *denotes that these two random variables are computationally indistinguishable; see Goldreich (2004), Ch. 3.2 for the standard notion of computational indistinguishability.*

### Fully homomorphic encryption

We use FHE (Rivest et al. 1978; Gentry 2009) as a key tool in our protocol. FHE supports encrypting messages and processing the resulting ciphertexts—without knowledge of the underlying messages—to obtain ciphertext for the results of computations on these underlying messages. For example, given two ciphertexts *c*_1_ and *c*_2_ encrypting messages *m*_1_ and *m*_2_ it is possible to produce ciphertexts *c*_Add_ and *c*_Mult_ so that decrypting these ciphertexts produces the messages *m*_1_ + *m*_2_ and *m*_1_ · *m*_2_, respectively. The ring in which the arithmetic operations are computed is called the *plaintext space*. We employ an FHE that supports, for any integer *N* ≥ 2, the plaintext space ℤ_*N*_, that is, the ring of integers modulo *N*, and where *N* is provided as input during key generation; we refer to this *N* as the *plaintext modulus*. More formally,

Definition 2 **(FHE).**

*A fully homomorphic encryption (FHE) scheme consists of four probabilistic polynomial time (aka, ppt) algorithms* E=(KeyGen,Enc,Dec,Eval) *with the following syntax:*

- KeyGen *takes as input a security parameter* λ *and an integer N* ≥ 2 *and outputs a key pair* $\left(pk,sk)\leftarrow KeyGen(1^{\lambda },N\right)$. *We assume without loss of generality that pk includes N in its description*.
- Enc *takes as input a public key pk and a message m* ∈ ℤ_*N*_*, and outputs a ciphertext* ctxt ← Enc(*pk*, *m*).
- Dec *takes as input a secret sk and a ciphertext* ctxt*, and outputs a plaintext message* $m^{\prime }\leftarrow Dec(sk,ctxt)$. *The correctness requirement is that for every* (*pk*, *sk*) *in the range of* KeyGen*, with overwhelming probability:* Dec(*sk*, Enc(*pk*, *m*)) = *m*.
- Eval *takes as input a public key pk, an arithmetic circuit C over* ℤ_*N*_ *accepting k inputs and producing l outputs for some k*, *l, and a vector of k ciphertexts*$\vec{ctxt}$*, and outputs a vector of l ciphertexts* $\vec{{ctxt}^{\prime }}\leftarrow Eval(pk,C;\vec{ctxt})$. *The* homomorphism *requirement is that for all* (*pk*, *sk*) *in the range of* KeyGen*, with overwhelming probability,*Dec(*sk*, Eval(*pk*, *C*; Enc(*pk*, *m*_1_), …, Enc(*pk*, *m*_*k*_))) = *C*(*m*_1_, …, *m*_*k*_)).

Compactness *says that the ciphertext size is independent of the class of supported homomorphic computations*. The semantic security *requirement is that for every λ*, *N*, *and m* ∈ ℤ_*N*_*, the joint distribution of pk (generated by* $\left(pk,sk)\leftarrow KeyGen(1^{\lambda },N\right)$*) and* ctxt ← Enc(*pk*, *m*) *is computationally indistinguishable from the joint distribution of pk and* ctxt_0_ ← Enc(*pk*, 0).

## Results

**Figure 2.**
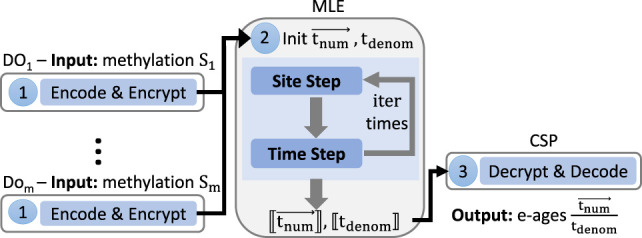
System flow: (1) Each DO_*j*_ encodes (in RNS) and encrypts her methylation values. (2) MLE initializes $\vec{t_{\mathrm{num}}},t_{\mathrm{denom}}$ and executes iter iterations of homomorphic evaluation of the site and time steps over encrypted values (written in double brackets [[⋅]]). (3) CSP decrypts, decodes, and outputs the resulting $\vec{t_{\mathrm{num}}},t_{\mathrm{denom}}$ values whose ratio is the predicted e-ages.

In this section, we introduce our privacy-preserving EPM protocol and our proof of concept system implementing it with its empirical evaluation (see an illustration of the system flow in Fig. 2 and a summary of the protocol in Supplemental Figs. S3, S5).

### Privacy-preserving EPM: protocol

We present three variants of our privacy-preserving EPM protocol. First, we describe a variant that preserves the privacy of the input values (observed methylation) but reveals the output (e-ages), and where these e-ages, which are rational numbers, are represented as a pair of integers—their numerator and denominator. Next, we describe the extension of this protocol to concealing both the input and the output numerators. We conclude by describing the third and final variant, which conceals both input and output (numerators and denominator), namely, it securely realizes the EPM functionality (Fig. 1).

The protocol is executed by the following parties: data owners DO_1_, …, DO_*m*_, where each DO_*j*_ has as input the observed methylation levels ${\hat{s}}_{1,j},\ldots ,\;{\hat{s}}_{n,j}$; a MLE server that performs the secure protocol computation; and a CSP who provides the public encryption and evaluation keys to the data owners and to the MLE, respectively, and decrypts the output. We remark that to simplify the presentation, we assume here that each DO holds methylation values for a single individual; nonetheless, each data owner may hold methylation values for an arbitrary number of individuals. Moreover, for simplicity of the presentation we assume that the CSP is the entity who decrypts; nonetheless, the CSP can provide the secret decryption key to any party (other than the MLE) who is authorized to decrypt and obtain the output. In this latter case, standard techniques must be employed to enroll and authenticate users, using proper credentials, to enforce the permissions policy.

All parties know the common parameters, which consist of the number of individuals *m*, the number of methylation sites *n*, the precision ℓ, an upper bound τ on the e-ages, and the employed homomorphic encryption scheme E=(KeyGen,Enc,Dec,Eval). The FHE E supports homomorphic computation with plaintext arithmetic over ℤ_*N*_ (i.e., where the message space consists of integers and the arithmetic operations—addition and multiplication—are computed modulo *N*). The plaintext modulus *N* is efficiently computed from the common parameter (see the formal details in Supplemental Fig. S4) and it is of size $O(iter\cdot (\ell +\log_{2}(mn\tau )))$.

The security and complexity guarantees of our protocol are specified in the following theorems. We use the $O_{\lambda ,\log N}()$ notation to hide complexity terms that are polynomial in λ and *N* and are determined solely by the complexity of the underlying encryption scheme E while being irrespective to our protocol, and denote by slots the number of data items that can be packed in each ciphertext. The proof is provided in Supplemental Notes 1–3.

Theorem 1 **(Security)**

*Our protocol securely realizes the EPM functionality* (Fig. 1*) against any passive computationally bounded adversary controlling any number of the data owners and at most one of MLE and CSP*.

Theorem 2 **(Complexity)**

*The complexity of our protocol is as follows*.

- *Each DO*_*j*_*’s runtime is dominated by the time to encrypt n methylation values, that is,* $O_{\lambda ,\log N}(n)$.
- *The CSP’s runtime is dominated by the time to decrypt the m (masked) output values, that is,* $O_{\lambda ,\log N}(m)$.
- *The MLE’s runtime is dominated by the time to homomorphically compute an arithmetic circuit of size* iter · *O*(*m*^2^ + *nm*) *and multiplicative depth* 3(iter − 1).
- *The communication volume is dominated by the size of the encrypted input, that is,* $O_{\lambda ,\log N}(mn)$.
- *The secure computing phase involves 1-round of interaction between the MLE and CSP (and is noninteractive, if revealing the common denominator of the output values is permitted)*.

### Our simplified protocol: hiding the input

Our input hiding EPM protocol is presented next (see also Supplemental Fig. S6). The protocol is composed of three phases—preprocessing, secure computing, and output postprocessing—as detailed next.

*Phase I: Preprocessing*. The preprocessing phase consists of key setup and data upload. Key setup is executed by the CSP who generates the required FHE key pairs and publishes the public keys. Data upload is executed by the DOs who format their methylation data, encrypt it, and pass the ciphertexts to the MLE for the secure computation. Data upload can occur over a period of time where methylation levels are gradually uploaded in encrypted form to cloud storage that will be made accessible to the MLE for secure computation when needed.

Elaborating on the above, our formatting includes the following three steps: first, rounding the data to ℓ decimal digits (for the ℓ specified in the common parameters); second, scaling the rounded values to integers (e.g., if ℓ = 2 and the methylation values are in (−1, 1) then scaling is via multiplying each value by 100); third, representing these integers in the residue number system (RNS) (Garner 1959), that is, mapping each integer *x* to the vector of its residues $\left(x\;\mathrm{mod}\;p_{1},\ldots ,x\;\mathrm{mod}\;p_{k}\right)$ where the moduli *p*_*i*_ are coprime and satisfy that ∏ *p*_*i*_ is larger than all intermediate plaintext values arising in our protocol. Computing in the RNS representation is equivalent to computing over the integers by the isomorphism $Z_{\prod_{i}p_{i}}\equiv Z_{\;p_{1}}\times Z_{\;p_{2}}\times \ldots \times Z_{\;p_{k}}$ guaranteed by the CRT and by setting *N*:= ∏_*i*_*p*_*i*_ sufficiently large as to avoid overflow. RNS is frequently employed in the context of FHE to efficiently perform operations on large integers by decomposing them into small numbers that fit in machine words (64-bit integers), where the large numbers may be large coefficients of ciphertext polynomials (Gentry et al. 2012; Bajard et al. 2016; Cheon et al. 2018; Halevi et al. 2019) or large plaintext values (Akavia et al. 2019). We rely on RNS for the latter: The plaintext values arising in our empirical evaluation are large, up to 1800-bit, whereas the FHE library we employ supports up to 64-bit plaintext values, a gap that is resolved by relying on RNS representation. Concretely, we employ as the *p*_*i*_’s in our empirical evaluation: 60 prime numbers of size 30-bits each. The homomorphic computations in the RNS representation are conducted entry-by-entry, with plaintext modulus *p*_*i*_ for entry *i*. Following Akavia et al. (2019), we support this via black-box use of the underlying FHE scheme, as detailed next.

To support homomorphic computation over plaintext values in RNS representation, in the preprocessing phase the CSP generates *k* key pairs $\left(pk_{i},sk_{i})\leftarrow \mathrm{KeyGen}(1^{\lambda },p_{i}\right)$ for *i* = 1, …, *k* and publishes *pk*:= (*pk*_1_, …, *pk*_*k*_); the data owners encrypt each methylation value ${\hat{s}}_{ij}$ under all keys, producing vectors of ciphertext $\mathrm{Enc}(pk,{\hat{s}}_{ij}):=(\mathrm{Enc}(pk_{1},{\hat{s}}_{ij}),\ldots ,\mathrm{Enc}(pk_{k},{\hat{s}}_{ij}))$ that are passed to the MLE. Subsequently, in the computing phase, the MLE executes the homomorphic evaluation with respect to all keys and their corresponding ciphertexts $\vec{ctxt}=({\vec{ctxt}}_{1},\ldots ,{\vec{ctxt}}_{k})$ (in parallel); we write this in shorthand notation as: $\mathrm{Eval}(pk,C;\vec{ctxt}):=(\mathrm{Eval}(pk_{1},C;{\vec{ctxt}}_{1}),\ldots ,\mathrm{Eval}(pk_{k},C;{\vec{ctxt}}_{k}))$. Eventually, in the output postprocessing phase, the CSP receives each result in encrypted RNS representation $\vec{ctxt^{\prime }}=({{ctxt}^{\prime }}_{1},\ldots ,{{ctxt}^{\prime }}_{k})$, and decrypts (in parallel), which we write in shorthand notation as $\mathrm{Dec}(sk,\vec{{ctxt}^{\prime }}):=\mathrm{Dec}(sk_{1},{{ctxt}^{\prime }}_{1}),\ldots ,\mathrm{Dec}(sk_{k},{{ctxt}^{\prime }}_{k})$, to obtain, in cleartext, the RNS representation $\left(o_{1},\ldots ,o_{k})\leftarrow \mathrm{Dec}(sk,\vec{{ctxt}^{\prime }}\right)$ of the output value. This RNS representation can then be efficiently transformed into the standard representation. For the sake of readability in the following (including in Supplemental Fig. S5), we simply write *pk*, *sk*, Enc(*pk*,·), Eval(*pk*, ·; ·), and Dec(*sk*, ·), while implicitly referring to computing with respect to all *k* keys (in parallel).

*Phase II: Secure computing*. In the secure computing phase the MLE employs homomorphic evaluation to produce a ciphertext encrypting the estimated e-ages, and publishes those ciphertexts. The homomorphic computation produces the same e-ages as in the EPM algorithm (cf. Supplemental Fig. S2), but includes important modifications that bypass complexity bottlenecks associated with homomorphic computation. To provide details, first recall that the EPM algorithm executes several iterations, each consisting of a *site step*, in which matrix inversion is computed to solve a linear-regression problem recovering latent parameters (called the rate $\vec{r}$ and the methylation at birth $\vec{s^{0}}$), and a *time step*, in which division is computed to update the e-ages $\vec{t}$ using these latent parameters. Both matrix inversion and division suffer from poor efficiency when computed homomorphically, and our solution avoids both, as follows. First, to avoid homomorphic matrix inverse we directly compute the solution to the regression problem using a closed-form algebraic solution from Snir (2020), Lemma 3. This formula however entails division, a complexity bottleneck that we avoid as discussed next. Second, to avoid homomorphic division we *represent rational numbers by the pair of integers: their numerator and denominator*, where we homomorphically update these (integral) values throughout the computation. That is, we represent rational numbers *x* ∈ ℚ by (*x*_num_, *x*_denom_) ∈ ℤ^2^ s.t. $x=\frac{x_{\mathrm{num}}}{x_{\mathrm{denom}}}$, and dividing *x* by an integer *Λ* is done by homomorphically updating its denominator to be *x*_denom_ · *Λ*. Alternatively, when we have a closed-form formula for *Λ*^−1^, we update the numerator to be *x*_num_ · *Λ*^−1^. These mathematical reformulations of the calculations, in both the site step and the time step phases, result in a formulation that exclusively involves addition, subtraction, and multiplication operations—which are efficient to compute homomorphically with FHE. We note that keeping track of the growing numerator and denominator, rather than computing division, leads to high magnitude numbers throughout the computation, which we reduce to a manageable scale using the CRT (as discussed above).

*Phase III: Output postprocessing*. In the output postprocessing phase, the CSP (or any authorized party that can fetch *sk* from CSP) decrypts and publishes the e-ages numerators and denominator (if the denominator is zero, output ⊥). Optionally: CSP performs division over the reals to obtain the cleartext e-ages in the standard representation of rational numbers.

### Our full protocol: hiding both input and output

For simplicity of the presentation the protocol described above reveals the entire vector of e-ages to all parties. It may be desired, however, to increase privacy so that each e-age (*t*_num,*j*_, *t*_denom_) is exposed in cleartext only to the corresponding data owner DO_*j*_, while being hidden from all other parties. Moreover, it may be desired to hide also the denominator *t*_denom_ and reveal to each DO_*j*_ only the ratio $t_{j}=\frac{t_{\mathrm{num},j}}{t_{\mathrm{denom}}}$ (where division is over the reals). This can be achieved via minor modifications. In what follows, we explain how to extend the protocol to hide also the output values $\vec{t_{\mathrm{num}}}$ and *t*_denom_, except for the designated output receiver. We first explain how to hide $\vec{t_{\mathrm{num}}}$, and then how to also hide *t*_denom_. Ciphertexts are denoted in bold font and their corresponding plaintext values in nonbold font, for example, Dec_sk_(***t***_denom_) = *t*_denom_. Concretely, the decrypted values of $\vec{t_{\mathrm{num}}^{\prime }}$, ***t***_num,*j*_, ***t***_denom_, $t_{\mathrm{denom}}^{\prime }$, $t_{\mathrm{denom}}^{-1}$, $\vec{t^{\prime }}$, ***t***_*j*_, mask_*j*_, mask^−1^ are, respectively: $\vec{t_{\mathrm{num}}^{\prime }}$, *t*_num,*j*_, *t*_denom_, $t_{\mathrm{denom}}^{\prime }$, $t_{\mathrm{denom}}^{-1}$, $\vec{t^{\prime }}$, *t*_*j*_, mask_*j*_, mask^−1^.

#### Hiding the numerators of the e-ages

Revealing each (*t*_num,*j*_, *t*_denom_) only to DO_*j*_ (and to no other party) is done as follows. In the data upload phase, each data owner DO_*j*_ samples a uniformly random mask mask_*j*_ ∈ ℤ_*N*_, encrypts it under *pk*, and sends the ciphertext to the MLE along with the encrypted methylation values. In the secure computing phase, before publishing the e-ages, the MLE homomorphically masks each e-ages numerators *t*_num,*j*_ by computing $t_{\mathrm{num},j}^{\prime }\leftarrow \mathrm{Eval}(pk,\mathrm{Add};t_{\mathrm{num},j},mask_{j})$ (where the function Add, given two integers, outputs their sum modulo *N*), and sends to the CSP these masked values $\vec{t_{\mathrm{num}}^{\prime }}$ along with ***t***_denom_. In the output postprocessing phase, the CSP decrypts and sends to each DO_*j*_ his masked output $\left(t_{\mathrm{num},j}^{\prime },t_{\mathrm{denom}}\right)$ in cleartext. Each DO_*j*_ then unmasks by computing $t_{\mathrm{num},j}=t_{\mathrm{num},j}^{\prime }-{mask}_{j}\;\mathrm{mod}\;N$ (in cleartext), and outputs $\frac{t_{\mathrm{num},j}}{t_{\mathrm{denom}}}$ where division is over the reals (output ⊥ if *t*_denom_ = 0).

#### Hiding also the denominator

The denominator *t*_denom_ is the same for all DO_*j*_’s, and so producing the output in the form of a pair of numerator and denominator cannot hide the denominator. When desired to hide also *t*_denom_, it can be done by adding the following further modifications to the above. In the secure computing phase, MLE does not publish $\left(\vec{t_{\mathrm{num}}},t_{\mathrm{denom}}\right)$ but rather does the following. MLE samples uniformly random $mask\in Z_{N}^{*}$ and homomorphically masks *t*_denom_ by $t_{\mathrm{denom}}^{\prime }\leftarrow \mathrm{Eval}(pk,\mathrm{Mult};t_{\mathrm{denom}},mask)$ (where Mult is the function that, given two integers, outputs their product modulo *N*), and sends the masked encrypted value $t_{\mathrm{denom}}^{\prime }$ to the CSP. The CSP then decrypts, computes (in cleartext) the inverse of $t_{\mathrm{denom}}^{\prime }$ in ℤ_*N*_ (except for outputting⊥, if $t_{\mathrm{denom}}^{\prime }=0$), denoted $t_{\mathrm{denom}}^{\prime -1}$, encrypts and sends the resulting ciphertext $t_{\mathrm{denom}}^{\prime -1}$ to MLE. In response, the MLE first homomorphically unmasks this inverse by computing $t_{\mathrm{denom}}^{-1}\leftarrow \mathrm{Eval}(pk,\mathrm{Mult};t_{\mathrm{denom}}^{\prime -1},{mask}^{-1})$ (where mask^−1^ is the inverse of mask in ℤ_*N*_, which MLE computes in cleartext); second, homomorphically computes $t_{j}\leftarrow \mathrm{Eval}(pk,\mathrm{Mult};t_{\mathrm{num},j},t_{\mathrm{denom}}^{-1})$, for each *j* ∈ [*m*]; third, homomorphically masks the resulting values with the mask_*j*_ received from the data owners by computing $t_{j}^{\prime }\leftarrow \mathrm{Eval}(pk,\mathrm{Add};t_{j},mask_{j})$, and sends the resulting masked ciphertexts $\vec{t^{\prime }}$ to the CSP. In the output postprocessing phase, the CSP decrypts, and, for each *j* ∈ [*m*], sends $t_{j}^{\prime }$ to DO_*j*_. Subsequently, for each *j*, DO_*j*_ unmasks to produce $t_{j}\leftarrow t_{j}^{\prime }-{mask}_{j}$, and then computes rational reconstruction (Wang et al. 1982; Fouque et al. 2002) to produce the rational number that is equal to computing $\frac{t_{\mathrm{num},j}}{t_{\mathrm{denom}}}$ over the reals.

### System and empirical evaluation

To assess the precision and efficiency of the secure protocol we proposed, we put it into practice as a proof of concept. An overview of our system flow is depicted in Figure 2.

#### Data set

Our experiments on real data obtained from the NCBI Gene Expression Omnibus (GEO; https://www.ncbi.nlm.nih.gov/geo/) under accession number GSE74193 (Jaffe et al. 2016) describe DNA methylation patterns in the frontal cortex during development. This methylation profile was used in Snir et al. (2019) to demonstrate logarithmic growth of human epigenetic aging along the life span.

The data set consists of 472 individuals and 7496 methylation sites. Chronological ages ranged between 0 and 99, and methylation values are floating-point numbers in (0, 1). In addition, as real data sets comprising of large numbers of individuals were not available to us, we ran some experiments on synthetic data generated based on the model in Snir (2020) (details appear in Supplemental Note 5; Supplemental Figs. S7, S8).

#### Data preparation

We conducted an initial feature selection utilizing Pearson's Correlation, a standard technique in machine learning. The process involved removing sites from the training data that displayed low correlation with the target ages. We found that an 80% correlation coefficient led to the selection of 716 sites for our experiments.

**Figure 3.**
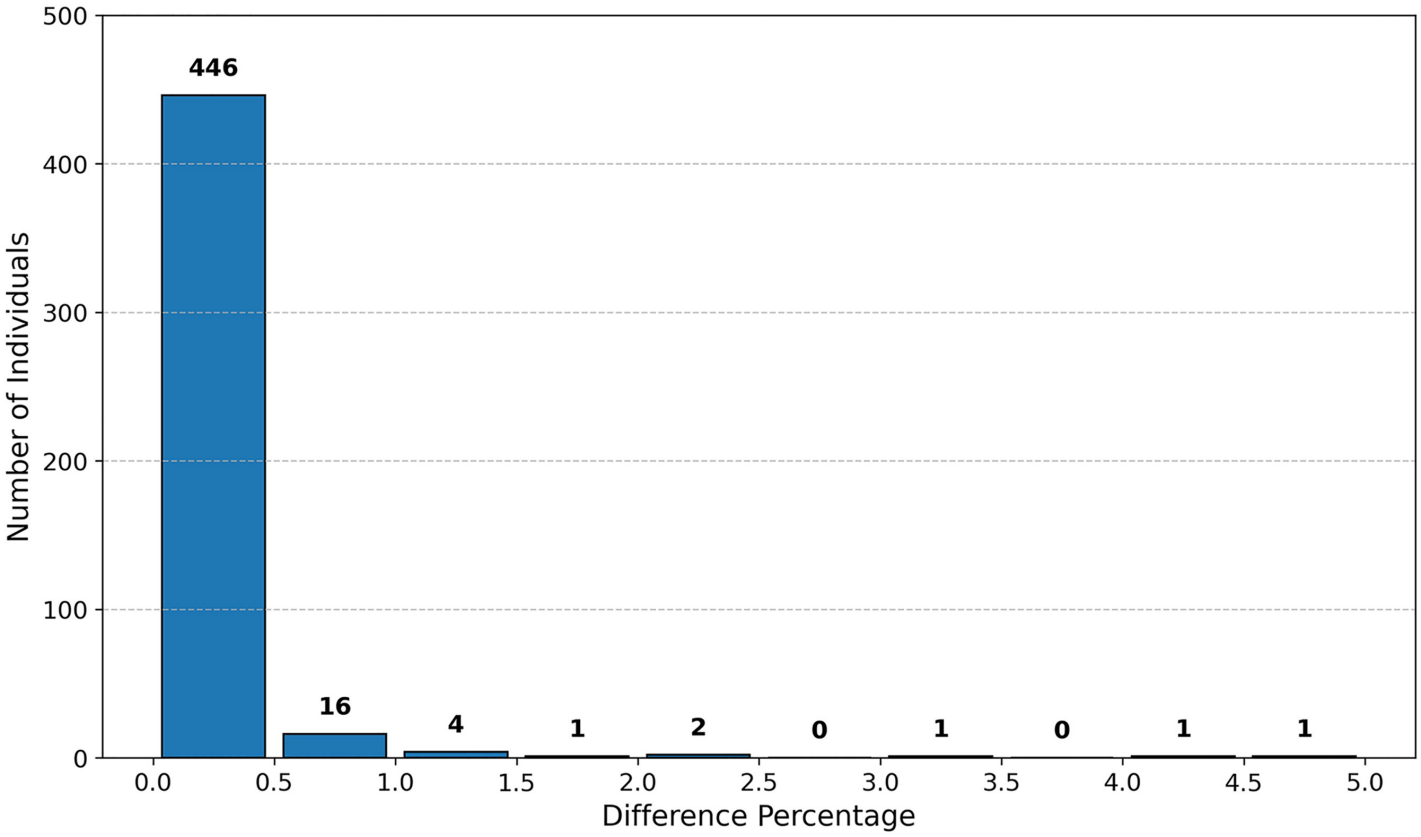
e-age difference percentage in our protocol versus EPM over cleartext.

The methylation and age values in the data set are in floating-point representation, whereas our selected FHE scheme—Brakerski/Fan–Vercauteren (BFV) (Brakerski 2012; Fan and Vercauteren 2012)—supports only integer data type. We, therefore, converted these data values to integers via rounding and scaling. To minimize the loss of accuracy, we conducted tests using various rounding values, indicating that rounding to two digits in the fractional part maintains good accuracy: no more than 0.5% loss in the vast majority of individuals (cf. Fig. 3). We require two additional digits for the integral part (for representing the ages, up to 99). The rounded numbers are then scaled to integers in the range [0, 10, 000). We then embed these integers in the ring of integers modulo *N*, for *N* sufficiently large as specified in Supplemental Note 4 and Supplemental Figure S4. We use the standard embedding, where positive integers $v\in [0,\frac{N}{2})$ are mapped to themselves, whereas negative integers $v\in [-\frac{N}{2},0)$ are mapped to $(N-v)\in [\frac{N}{2},N)$.

#### Software libraries and hardware

We implemented our proof of concept system in Python version 3.10.12 using Pyfhel version 3.4.1 (Ibarrondo and Viand 2021) implementation for the BFV FHE scheme, sympy version 1.12 (Meurer et al. 2017) for CRT, and numpy version 1.24.3 (Harris et al. 2020) for matrix storage and operations. Our system was executed on an AWS cloud server featuring 96 Intel Xeon 8275CL CPUs, 192 GB of RAM, and Ubuntu Server 22.04 OS.

#### Encryption parameters

FHE has several parameters that require configuration to match various use cases. Throughout the implementation phase, we conducted a thorough assessment of various parameter configurations and encryption schemes, ultimately arriving at the following set of parameters, which align well with the volume of mathematical operations and data dimensions used in our implementation and testing: 16k polynomial modulus degree, 30-bit plaintext modulus prime numbers, and a security key size of 128 bits.

Furthermore, our implementation leverages the packing feature, an integral component of FHE encryption. This feature enables the encryption of multiple cleartext values within a single encrypted context object, granting us the capability to perform mathematical operations on all values in parallel. This provides a significant increase in calculation efficiency in cases of vector multiplication and addition operations as well as calculation of several individual values in parallel as required in both the site and time steps. Pyfhel aligns the packed array size to the polynomial modulus degree, encompassing 16k individual cells in a 2 × 8k matrix format. As the method for accessing the second row of cells in the matrix did not conform well with our implementation requirements, we utilized only the first 8k of cells. Required changes have since been added to Pyfhel and can be a point for improvement of our implementation in the future.

#### Multiplicative depth

To guarantee the security of FHE, each ciphertext must contain a certain level of noise. Mathematical operations, particularly multiplication, contribute significantly to noise accumulation. If the noise surpasses a predefined threshold, it results in an overflow, rendering the ciphertext indecipherable. The threshold is determined by encryption context parameters, with the polynomial modulus degree and plaintext modulus value being the most influential. Our protocol's analysis determined the maximum number of consecutive multiplications, termed *multiplicative depth*, performed on a ciphertext within a single iteration. Our analysis revealed a multiplicative depth of 3 for the calculation of ***t***_num_ which increases with each iteration of the protocol. This prompted exploration for noise mitigation solutions. Although increasing the polynomial modulus degree is an option, it adversely affects performance and memory requirements. An alternative is *bootstrapping*, a noise reduction operation. Although time-consuming, if applied selectively, it minimally impacts the overall protocol execution time. Our analysis suggested that applying bootstrapping to ***t***_num_ and ***t***_denom_ at the end of the time step should support any amount of iterations. Unfortunately, our chosen FHE library lacked bootstrapping support, leading us to simulate it through a decrypt–re-encrypt process with a trusted third party. This approach is for testing purposes only and falls short of meeting our strict security requirements. Future authentic bootstrapping support is essential for securely implementing the protocol. Meanwhile, bootstrapping tests in C++ using HElib (Halevi and Shoup 2014) yielded a 159-second execution time for a single 16k polynomial modulus degree which must be considered as part of the overall protocol execution time.

#### Parallel computation

The use of RNS in our protocol entails multiple algorithm runs, each with a different prime number for the plaintext modulus. Concretely, our system employs 60 prime numbers, of size 30 bits each, to support the 1800-bit plaintext values arising in our computation. We conducted a performance evaluation by executing three iterations of the protocol with a single prime number, revealing a total runtime of 45 minutes. To mitigate the substantial increase in runtime that would arise from running with multiple prime numbers in serial, we implemented parallel multiprocess computation. In this setup, each process is responsible for the computation associated with a single prime number.

#### Accuracy

We performed a comparative accuracy analysis comparing the ages computed by our secure protocol to those obtained through our implementation of the cleartext algorithm, exhibiting that the maximum and average differences are 0.18 and 0.04 years, respectively (with standard deviation 0.03 years). We also measure the *difference percentage* defined, for each individual *i*, to be: $\left(\frac{\left|t_{i}^{\mathrm{EPM}}-t_{i}^{\mathrm{Our}}\right|}{t_{i}^{\mathrm{EPM}}}\right)\cdot 100$ where $t_{i}^{\mathrm{EPM}}$ and $t_{i}^{\mathrm{Our}}$ are the ages calculated for individual *i* by the cleartext algorithm and our secure protocol, respectively. The results (depicted in Fig. 3) show that for 446 out of 472 individuals, the difference percentage is 0.5% or less; for an additional 16 individuals, it is between 0.5% and 1%; and for the remaining 10 individuals, it is between 1% and 5%. Our full e-age comparison results are available in Supplemental Table S1.

Furthermore, to evaluate the number of individuals required for achieving high accuracy in e-age prediction of the cleartext EPM algorithm, we ran experiments on a synthetic data set with up to 20,000 individuals (cf. 472 individuals in the real data set on which we conducted all other experiments). Our experiments suggest that a sample size of ∼5000 individuals is sufficient for accurate e-age prediction (concretely, with RSS <1).

#### Runtime

We used 60 prime numbers for the computation, thereby concurrently executing 60 parallel processes, each running three iterations (i.e., setting iter=3) of homomorphic site-step and time-step computation, resulting in an observed execution time of 3 hours and 7 minutes. To ascertain an accurate depiction of the time required for the data set under consideration, we must incorporate the estimated time of the bootstrapping operations. As we expected additional overhead due to parallel execution, we tested 60 parallel bootstrap operations and observed a total runtime of 282 seconds. As there are four bootstrap operations in three iterations of the protocol, we added 4 × 282 seconds to the total expected execution time which amounts to: 3 hours, 25 minutes, and 48 seconds.

**Figure 4.**
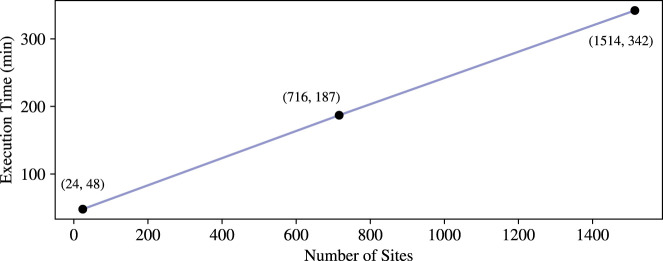
Computing phase runtime (Our) per number of sites.

Furthermore, we measure how execution timescales in the number of sites, by measuring the runtime of our protocol also on 24 sites (as in Goldenberg et al. 2022) and 1514 sites, all with 472 individuals. The results show a runtime that grows linearly in the number of sites, as depicted in Figure 4.

**Figure 5.**
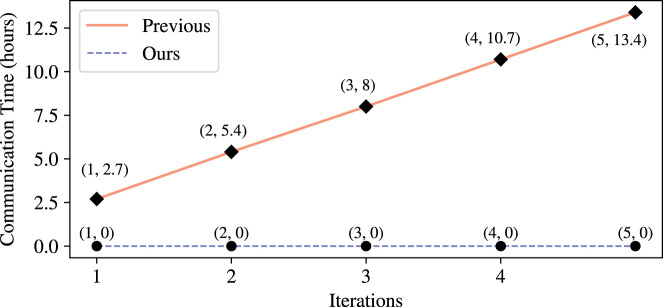
Computing phase communication time (estimate) (Goldenberg et al. 2022) versus Our.

Finally, to evaluate the runtime improvement we offer, when compared to the prior secure EPM, we estimate the communication time between the two servers required for executing the protocol of Goldenberg et al. (2022): In each iteration their communication entails the MLE sending an encrypted 2*n* × 2*n* matrix and receiving from the CSP an encrypted 2*n*-dimensional vector, that is, transmitting a total of iter · (4*n*^2^ + 2*n*) encrypted values. As their protocol was not fully implemented, we must complement some implementation details to estimate their performance. First, we suppose their implementation employs FHE supporting packing SLOTS = 16k cleartext values in each ciphertext, to have equal footing with the packing parameter in our implementation. We then calculated the size in bytes of a ciphertext with 16k slots, which resulted in a value of 2 MB. Second, we suppose they utilize packing as recommended in the work (Akavia et al. 2019) they rely on, implying that they transmit $\left(\frac{{\left(2n\right)}^{3}}{\mathrm{SLOTS}}+(2n)\right)$ ciphertexts in each iteration (cf. Akavia et al. 2019, Section 5.2.1, last paragraph). Third, we suppose their communication is over a wide area network (WAN) of a typical speed (150 Mbps); the focus on WAN follows by supposing they would mount their two servers on separate cloud service providers (e.g., AWS vs. Google Cloud) to increase the plausibility of their noncollusion assumption. Supposing all the above, the expected communication time of Goldenberg et al. (2022), when executed on the same number of sites, individuals, and iterations as in our protocol (i.e., *n* = 716, *m* = 472, and iter = 3), is 8 hours. Further results are depicted in Figure 5.

## Discussion

We presented a new privacy-preserving protocol for computing the EPM model over federated data. Our protocol offers better asymptotic communication complexity and stronger privacy than the prior work (Goldenberg et al. 2022) as well as faster concrete efficiency (see Table 1). The uniqueness of the direction pursued here stems from the fact that privacy in the context of epigenetics, as opposed to GWAS, was rarely studied before, only a single work handling gene expression data (Akavia et al. 2024) preceded our direction of epigenetic aging as pursued here. Moreover, the processing done here is not over sequences, DNA, or protein, and hence borrows tools from other areas from the latter.

**Table 1.** Computing phase in Goldenberg et al. (2022) (Prev) versus Ours. *m*, *n*, iter, and (hom.) ops. denote the number of individuals, methylation sites, iterations, and (homomorphic) operations

	Output hiding		Communication		Complexity	
	$\vec{t_{\mathrm{num}}}$	*t*_denom_	Rounds	#Ctxt	MLE (hom.ops.)	CSP (ops.)
Prev	×	×	iter	iter ·*O*(*n*^2^)	iter ·*O*(*n*^2^ + *nm*)	iter ·*O*(*n*^3^)
Our_1_	×	×	0	0	iter ·*O*(*m*^2^ + *nm*)	0
Our_2_	✓	×				
Our_3_	✓	✓	1	*O*(1)		*O*(1)

The approach presented here can be generalized. Our solution solves an optimization problem over degree-two polynomials which is not necessarily solvable in polynomial time in the general case. In the upper level of our solution, the variable set is decomposed such that we can apply an EM approach over every part of the variable set in an alternating manner. EM in general is very widespread in biology (Do and Batzoglou 2008) with applications in areas such as the study of quantitative trait loci (QTL) (Xu 2010) or protein structure (Ward et al. 2021). Our novel approach, that simplifies the handling of every part of the variable set, and hence allows applying encryption algorithms, may open the way also to these areas in computational biology where privacy is mandatory (e.g., QTL).

### Software availability

Our code is available as open source under MIT license at GitHub (https://github.com/ASEC-lab/EPM-code) and as Supplemental Code.

## Supplementary Material

Supplement 1

Supplement 2

Supplement 3

Supplement 4
