# Supplementary material for: Privacy-preserving biological age prediction over federated human methylation data using fully homomorphic encryption
Source: Genome Res. 2024 Sep;34(9):1324–33. doi: 10.1101/gr.279071.124 (PMC11529865; doi:10.1101/gr.279071.124)
Supplement: Supplement 2 [file Supplemental_Figures.pdf]

Supplemental Material for: "Privacy Preserving Epigenetic PaceMaker Stronger Privacy and Improved Efficiency"

Meir Goldenberg, Loay Mualem, Amit Shahar, Sagi Snir, and Adi Akavia[0000-0003-0853-3576]

University of Haifa, Israel
meirgold@hotmail.com, loaymua@gmail.com, ashaha16@campus.haifa.ac.il, ssagi@research.haifa.ac.il, adi.akavia@gmail.com

Table of Contents:

1. Supplemental Figure S1: X, β, and y
2. Supplemental Figure S2: EPM Algorithm on cleartext data
3. Supplemental Figure S3: Notations summary
4. Supplemental Figure S4: Algorithm for finding a lower bound of the plaintext modulus
5. Supplemental Figure S5: Our Secure EPM Protocol
6. Supplemental Figure S6: Simplified EPM Functionality
7. Supplemental Figure S7: Synthetic data generation and simulation
8. Supplemental Figure S8: RSS vs number of individuals in the dataset.

$$X = \left[\begin{array}{cccc|cccc}
t_1 & 0 & \dots & 0 & 1 & 0 & \dots & 0 \\
t_2 & 0 & \dots & 0 & 1 & 0 & \dots & 0 \\
 & & \vdots & & & \vdots & & \\
t_m & 0 & \dots & 0 & 1 & 0 & \dots & 0 \\
0 & t_1 & \dots & 0 & 0 & 1 & \dots & 0 \\
0 & t_2 & \dots & 0 & 0 & 1 & \dots & 0 \\
 & & \vdots & & & \vdots & & \\
0 & t_m & \dots & 0 & 0 & 1 & \dots & 0 \\
 & & \vdots & & & \vdots & & \\
 & & \vdots & & & \vdots & & \\
0 & \dots & 0 & t_1 & 0 & \dots & 0 & 1 \\
0 & \dots & 0 & t_2 & 0 & \dots & 0 & 1 \\
 & & \vdots & & & \vdots & & \\
0 & \dots & 0 & t_m & 0 & \dots & 0 & 1
\end{array}\right], \beta = \begin{bmatrix} r_1 \\ \vdots \\ r_n \\ - \\ s_1^0 \\ \vdots \\ s_n^0 \end{bmatrix}, Y = \begin{bmatrix} \hat{s}_{1,1} \\ \hat{s}_{1,2} \\ \\ \vdots \\ \\ \hat{s}_{n,m} \end{bmatrix}$$

Supplemental Figure S1: X, β, and y

Parameters: Number individuals m, sites n, and iterations iter.

Input: A matrix $\hat{S} = \hat{s}_{i,j}$ holding observed methylation levels for on all sites $i \in [n]$ and for each individual $j \in [m]$.

Output: $t_1, \ldots, t_m$

Steps:

1. **Initialization:** Initialize $t_1, \ldots, t_m$ to random age values and $\mathsf{isFail} = \mathsf{FALSE}$. Let X be the $mn \times 2n$ matrix associated with t_js as specified in Figure S1. Let y be a mn-dimension vector holding the entries of $\hat{S}$ from top down, left to right (i.e. $y_{im+j} \leftarrow \hat{s}_{i,j}$)
2. For iter iterations do:
   (a) **Site step:** Solve the linear regression problem on input (X, y), denote the solution by the length $2n$ vector $\beta = (r_1, \ldots, r_n, s_1^0, \ldots s_n^0)$ (cf. Figure S1)
   (b) **Time step:** For each $j \in [m]$, calculate
   $$t_j \leftarrow \frac{\sum_i r_i(\hat{s}_{i,j} - s_i^0)}{\sum_i r_i^2} \tag{1}$$
   unless the denominator is zero in which case we set t_j to an arbitrary value and set $\mathsf{isFail} = \mathsf{TRUE}$.
3. If $\mathsf{isFail} = \mathsf{FALSE}$ return $(t_1, \ldots, t_m)$ (else return $\perp$).

Supplemental Figure S2: EPM Algorithm on cleartext data

- m and n are the number of individuals and methylation sites respectively.
- $\hat{s}_{i,j}$ is the observed methylation at individual j and site i.
- $\overrightarrow{t} = (t_1, \ldots, t_m)$ are the e-ages as updated throughout the computation, where each t_j is represented as a pair $(t_{\mathsf{num},j}, t_{\mathsf{denom}})$ of its numerator and denominator (initialize to $(t_{\mathsf{num},j}, t_{\mathsf{denom}}) = (t_j, 1)$).
- ψ_k and ϕ_k (for $k \in [m]$) are defined by:

$$\psi(t_{\mathsf{num},k}, t_{\mathsf{denom}}) = (-m \cdot t_{\mathsf{num},k} + \sum_{j \leq m} t_{\mathsf{num},j}) \cdot t_{\mathsf{denom}} \tag{2}$$

$$\phi(t_{\mathsf{num},k}) = t_{\mathsf{num},k} \sum_{j \leq m} t_{\mathsf{num},j} - \sum_{j \leq m} t^2_{\mathsf{num},j} \tag{3}$$

- The $n \times nm$-dimensional matrices U, L and nm-dimensional vector Y are defined by:

$$U(\overrightarrow{t_{\mathsf{num}}}, t_{\mathsf{denom}}) = \begin{bmatrix} \psi_1 \cdots \psi_m & 0 & \cdots & & 0 \\ 0 \cdots 0 & \psi_1 \cdots \psi_m & 0 & \cdots \cdots & 0 \\ \vdots & & & & \vdots \\ 0 & \cdots & & 0 & \psi_1 \cdots \psi_m \end{bmatrix} \tag{4}$$

$$L(\overrightarrow{t_{\mathsf{num}}}) = \begin{bmatrix} \phi(t_1) \cdots \phi(t_m) & 0 & \cdots & & 0 \\ 0 \cdots 0 & \phi(t_1) \cdots \phi(t_m) & 0 & \cdots \cdots & 0 \\ \vdots & & & & \vdots \\ 0 & \cdots & & 0 & \phi(t_1) \cdots \phi(t_m) \end{bmatrix} \tag{5}$$

$$Y = (\hat{s}_{1,1}, \ldots, \hat{s}_{1,m}, \hat{s}_{2,1}, \ldots, \hat{s}_{2,m}, \ldots, \hat{s}_{n,1}, \ldots, \hat{s}_{n,m},)^T \tag{6}$$

- $\mathsf{MatVecMult}$ is the function that given a matrix and a vector (of matching dimensions) returns their product.
- $\overrightarrow{r} = (r_1, \ldots, r_n)$ and $\overrightarrow{s^0} = (s^0_1, \ldots, s^0_n)$ are sites' rates and methylation at birth values, respectively, as computed throughout the EPM algorithm; and $\overrightarrow{r'}, \overrightarrow{s'^0}$ their scaling by the factor Λ^{-1} defined by:

$$\Lambda^{-1}(\overrightarrow{t_{\mathsf{num}}}) = \left(\sum_{j \leq m} t_{\mathsf{num},j} \right)^2 - m \sum_{j \leq m} t^2_{\mathsf{num},j} \tag{7}$$

- The e-age are updated (in each time step) according to the following formulae:

$$t_{\mathsf{num}}(\Lambda^{-1}, \overrightarrow{r'}, \overrightarrow{s'^0}, (\hat{s}_{i,j})_{i \in [n]}) = \sum_{i \leq n} r'_i \left(\Lambda^{-1} \hat{s}_{i,j} - {s'}^0_i \right) \tag{8}$$

$$t_{\mathsf{denom}}(\overrightarrow{r'}) = \sum_{i \leq n} {r'_i}^2 \tag{9}$$

Supplemental Figure S3: Notations summary: inputs, variables, and functions to be homomorphically evaluated in our protocol.

Input: Positive integers $m, n, \mathsf{iter}, \ell$ (the parameters of the EPM functionality).

Output: A positive integer N_0 (a lower bound on the plaintext modulus N for the privacy preserving EPM protocol)

Steps:

1. Initialize: $\alpha \leftarrow 1$, $\sigma \leftarrow 10^\ell$, $\tau \leftarrow 100 \cdot 10^\ell$
2. For iter iterations do:
   (a) $N_0 \leftarrow n \cdot 6m^4 \cdot \tau^3 \cdot \sigma^2 \cdot \alpha$
   (b) $\tau \leftarrow N_0$
   (c) $\alpha \leftarrow n \cdot 4m^4 \cdot \tau^2 \cdot \sigma^2 \cdot \alpha^2$
3. $N_0 \leftarrow N_0 \cdot 2$
4. Return N_0

Supplemental Figure S4: Algorithm for finding a lower bound of the plaintext modulus. For simplicity we assume that the upper age bound is 100 and methylation values are in range of (0, 1).

Parties: Data Owners $\mathsf{DO}_1, \ldots \mathsf{DO}_m$, Machine Learning Server MLE and Crypto Service Provider CSP.

Common Parameters: the number of data owners/individuals m and methylation sites n, the precision ℓ, the number of iterations iter, a fully homomorphic encryption scheme $\mathcal{E} = (\mathsf{KeyGen}, \mathsf{Enc}, \mathsf{Dec}, \mathsf{Eval})$, and a security parameter λ. Let N be an integer s.t. $N \geq N_0$, where computations are in the ring $\mathbb{Z}_N$ of integers modulo the N (unless explicitly stated otherwise).

Input: Each DO_j holds methylation values $(\hat{s}_{i,j})_{i\in[n]} \in (0,1)^n$ (MLE and CSP have no input).

Output: Predicted epigenetic ages $t_1, \ldots, t_m$

I. Pre-processing phase (key generation and data upload):

1. **Setup:** The CSP executes $(pk, sk) \leftarrow \mathsf{KeyGen}(1^\lambda, N)$ and publishes the public key pk.
2. **Data upload:** Each DO_j rounds her input to ℓ decimal digits, encrypts the rounded values with the public key pk, and sends the ciphertexts to the MLE.

II. Secure computing Phase: MLE, upon receiving ciphertexts $\hat{\boldsymbol{s}}_{i,j}$ for the methylation values $\hat{s}_{i,j}$ $(i \in [n], j \in [m])$, initializes the e-ages t_j to uniformly random in $[0, \tau]$ where τ (for τ as set in Figure S4) represented as a numerator and denominator pair $(t_{\mathsf{num},j}, t_{\mathsf{denom}}) = (t_j, 1)$, and does the following:

1. For iter iterations do:
   (a) **Site step:** Homomorphically solve the linear system of the EPM site step (Figure S2, Step 2a) as follows:
      i. Implicitly construct $\boldsymbol{U}, \boldsymbol{L}$ and $\boldsymbol{Y}$ by homomorphically computing:

$$\boldsymbol{\psi}_k \leftarrow \mathsf{Eval}(pk, \psi; \boldsymbol{t}_{\mathsf{num},k}, \boldsymbol{t}_{\mathsf{denom}})$$
$$\boldsymbol{\phi}_k \leftarrow \mathsf{Eval}(pk, \phi; \boldsymbol{t}_{\mathsf{num},k})$$

      and using $(\hat{\boldsymbol{s}}_{i,j})_{i\in[n],j\in[m]}$, respectively. We remark that $\overrightarrow{t_{\mathsf{num}}}$ is in cleartext form at the 1st iteration, and in encrypted form in subsequent ones.
      ii. Homomorphically compute ciphertexts encrypting the numerator of the linear system's solution:

$$\overrightarrow{\boldsymbol{r'}} \leftarrow \mathsf{Eval}(pk, \mathsf{MatVecMult}; \boldsymbol{U}, \boldsymbol{Y})$$
$$\overrightarrow{\boldsymbol{s'^0}} \leftarrow \mathsf{Eval}(pk, \mathsf{MatVecMult}; \boldsymbol{L}, \boldsymbol{Y})$$

      and the corresponding denominator: $\boldsymbol{\Lambda^{-1}} \leftarrow \mathsf{Eval}(pk, \Lambda^{-1}; \overrightarrow{\boldsymbol{t}_{\mathsf{num}}})$.
   (b) **Time step:** Homomorphically update the e-ages t_j (cf. Figure S2, Step 2b) by computing their updated numerators and denominator:

$$\boldsymbol{t}_{\mathsf{num},j} \leftarrow \mathsf{Eval}(pk, t_{\mathsf{num}}; \boldsymbol{\Lambda^{-1}}, \overrightarrow{\boldsymbol{r'}}, \overrightarrow{\boldsymbol{s'^0}}, (\hat{\boldsymbol{s}}_{i,j})_{i\in[n]})\ \forall j \in [m]$$
$$\boldsymbol{t}_{\mathsf{denom}} \leftarrow \mathsf{Eval}(pk, t_{\mathsf{denom}}; \overrightarrow{\boldsymbol{r'}})$$

2. Publish the ciphertexts for the updated e-ages: $\boldsymbol{t}_{\mathsf{num},1}, \ldots, \boldsymbol{t}_{\mathsf{num},m}$ and $\boldsymbol{t}_{\mathsf{denom}}$.

III. Post processing phase: The CSP decrypts to obtain $t_{\mathsf{num},j} \leftarrow \mathsf{Dec}(sk, \boldsymbol{t}_{\mathsf{num},j})$, for all $j \in [m]$, and $t_{\mathsf{denom}} \leftarrow \mathsf{Dec}(sk, \boldsymbol{t}_{\mathsf{denom}})$, and publish the cleartext e-ages' numerators and denominator $(\vec{t}_{\mathsf{num}}, t_{\mathsf{denom}})$. These values represent the e-ages: $t_1 \leftarrow \frac{t_{\mathsf{num},1}}{t_{\mathsf{denom}}}, \ldots, t_m \leftarrow \frac{t_{\mathsf{num},m}}{t_{\mathsf{denom}}}$ where division is over the reals (output $\perp$, if $t_{\mathsf{denom}} = 0$).

Supplemental Figure S5: Our Secure EPM Protocol. See Supplemental Figure S3 for the definition of the functions $\Lambda^{-1}, \psi, \phi, \mathsf{MatVecMult}, t_{\mathsf{num}}, t_{\mathsf{denom}}$, matrices U, L, vector Y, and the bounds τ and N_0. Ciphertexts are denoted in **Boldface**, i.e., $\overrightarrow{\boldsymbol{t}_{\mathsf{num}}}, \boldsymbol{\Lambda^{-1}}, \boldsymbol{t}_{\mathsf{denom}}, \boldsymbol{U}, \boldsymbol{L}, \boldsymbol{Y}, \overrightarrow{\boldsymbol{r'}}$ and $\overrightarrow{\boldsymbol{s'^0}}$ decrypt to $\overrightarrow{t_{\mathsf{num}}}, \Lambda^{-1}, t_{\mathsf{denom}}, U, L, Y, \overrightarrow{r'}$ and $\overrightarrow{s'^0}$ respectively. We note that Λ^{-1} denotes both a function and its output $\Lambda^{-1}(\vec{t}_{\mathsf{num}})$.

Parties: Data-Owners $\mathsf{DO}_1, \ldots, \mathsf{DO}_m$, a Machine Learning Engine (MLE), and a Crypto Service Provider (CSP).

Common Parameters: The number of individuals m; the sites $s_1, \ldots, s_n$; the precision ℓ, where all values in $\mathbb{R}$ are scaled to $[-\delta, \delta]$ with a precision of ℓ digit; number of iterations iter. Denote by N the smallest sufficiently larger integer as to guarantee that no overflow occurs during the computation, i.e., we require that $N > N_0$ for N_0 as specified in Figure S4

Input: Each data owner j holds observed methylation levels $\hat{s}_{1,j}, \ldots, \hat{s}_{n,j}$.

Output: All parties receive as output the epigenetic age estimation $t_1, \ldots, t_m$ that are the output of the EPM algorithm (Figure S2) when executed on input $(\hat{s}_{i,j})_{i \in [n], j \in [m]}$ for iter iterations.

Leakage Profile: The common parameters and the outputted epigenetic ages t_j provided in the following representation. Each t_j is represented as a pair of integers whose ratio equals t_j, concretely, by the numerator and denominator computed in the Time Step at the final iteration of the EPM algorithm of Figure S2.

Supplemental Figure S6: EPM Functionality, simplified to reveal the output

I. Data preparation for N individuals:

1. **Rates (r) and methylation at birth (s^0):** We define $r_{min}, r_{max}, s^0_{min}, s^0_{max}$ to be the minimum and maximum rate and s^0 values derived by our cleartext algorithm as part of the empirical evaluation results on real data. For each methylation site i uniformly sample $r_i \in [r_{min}, r_{max}]$ and $s^0_i \in [s^0_{min}, s^0_{max}]$
2. **Ages:** We sample T as a uniformly generated list of chronological age values where for each $t_j \in T$ it holds that $0 \leq t_j \leq 99$
3. **Noise value:** We sample $\mathcal{E}_{i,j} \sim N(0, \sigma^2)$ as a normally distributed noise value where $\sigma = 0.15$ as used in [Snir et al., 2016]
4. **Methylation values:** We compute $\hat{s}_{ij}$ as the methylation value for individual j at site i derived using the formula $\hat{s}_{ij} = s^0_i + r_i t_i + \mathcal{E}_{i,j}$ as detailed in [Snir, 2020] using the age, rate, methylation at birth and noise values described above.
5. **Ground truth epigenetic age values:** We compute our ground truth epigenetic ages T_{gt} by running the EPM site step using the above methylation and age values as inputs.
6. **Dataset construction:** The chronological ages (T), methylation values ($\hat{s}_{ij}$) and ground truth epigenetic ages (T_{gt}) from above will serve as input to the next stage.

II. Simulation: Defining N as the total number of individuals in our synthetic dataset, run the following steps for the simulation:

1. Set $n \leftarrow 50$
2. Repeat while $n < N$
   (a) Derive a subset of n individuals and their methylation values from the full dataset, starting from the first individual.
   (b) Run the cleartext EPM algorithm as defined in S2 using the derived dataset for 3 iterations.
   (c) Let $T_{predicted}$ be the predicted ages outputted by the EPM algorithm.
   Calculate $RSS = \sum_{j \leq 50} (t_{predicted_j} - t_{gt_j})^2$ where $t_{predicted_j} \in T_{predicted}$ and $t_{gt_j} \in T_{gt}$
   (d) Add the calculated RSS to RSS_List
   (e) $n \leftarrow n + 50$
3. return RSS_List

Supplemental Figure S7: synthetic data generation and simulation

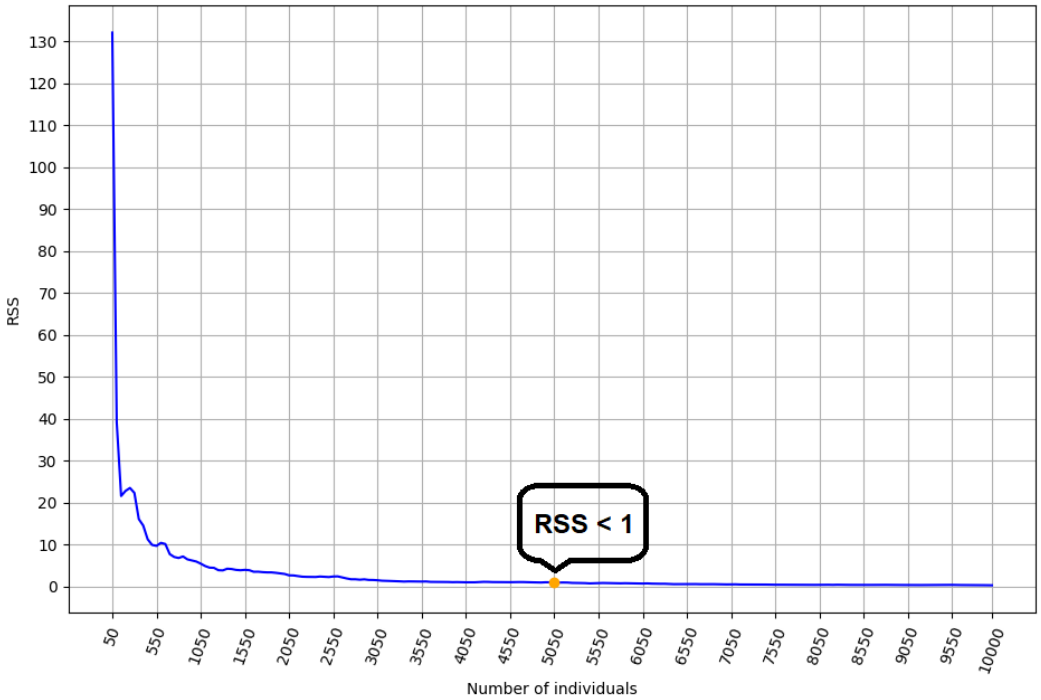

Supplemental Figure S8: RSS vs number of individuals in the dataset. RSS measurement is for the first 50 individuals.

References

Sagi Snir. Epigenetic pacemaker: closed form algebraic solutions. *BMC genomics*, 21(2):1–11, 2020.

Sagi Snir, Bridgett M vonHoldt, and Matteo Pellegrini. A statistical framework to identify deviation from time linearity in epigenetic aging. *PLoS computational biology*, 12(11):e1005183, 2016.
