# Supplementary material for: Privacy-preserving biological age prediction over federated human methylation data using fully homomorphic encryption
Source: Genome Res. 2024 Sep;34(9):1324–33. doi: 10.1101/gr.279071.124 (PMC11529865; doi:10.1101/gr.279071.124)
Supplement: Supplement 3 [file Supplemental_Notes.pdf]

Supplemental Material for: "Privacy Preserving Epigenetic PaceMaker Stronger Privacy and Improved Efficiency"

Meir Goldenberg, Loay Mualem, Amit Shahar, Sagi Snir, and Adi Akavia[0000−0003−0853−3576]

University of Haifa, Israel
meirgold@hotmail.com, loaymua@gmail.com, ashaha16@campus.haifa.ac.il, ssagi@research.haifa.ac.il, adi.akavia@gmail.com

Table of Contents:

1. Supplemental Note 1: Proof of Theorem 1, Correctness
2. Supplemental Note 2: Proof of Theorem 1, Privacy
3. Supplemental Note 3: Proof of Theorem 2, Complexity
4. Supplemental Note 4: Proof of Maximum Plaintext Modulus Value
5. Supplemental Note 5: EPM Quantitative Insights

Supplemental Note 1: Proof of Theorem 1, Correctness

Proof (of Theorem 1, Correctness). We hereby sketch the main idea to prove that the ciphertext produced by the MLE upon the termination of the secure computing phase (hencefoth, the secure algorithm) decrypt to the same value as produced by executing the algorithm of Snir [Snir, 2020] (cf. Supplemental Figure S2) on cleartext values (hencefoth, the cleartext algorithm).

We note that all calculations in the cleartext algorithm are performed over the real numbers while the secure algorithm performs calculations modulo N (after proper rounding and scaling of the input), where we assume that N is sufficiently large so that no value exceeds $N/2$. The correctness proof is w.r.t to the cleartext algorithm when executed on the values with the same scaling and rounding.

By the correctness of the FHE scheme (cf. Section on Fully Homomorphic Encryption), the computations performed homomorphically over encrypted data decrypt to the same value as if applying those computations on the underlying cleartext values. Therefore, it suffices to prove that the latter has the same outcome as the cleartext algorithm. This is not immediate, because the homomorphic computations entail modifications that we introduced to the cleartextg algorithm to avoid complexity bottlenecks. Yet, as we argue below, indeed this is the case.

Snir [Snir, 2020, Lemmas 2 and 3] describes two matrices $[U]_{k,l}$ and $[L]_{k,l}$ of structure similar to our U, L (see Supplemental Figure S3) except that their non zero diagonal elements are defined to be:

$$\psi_{snir}(k) = \Lambda \cdot (-m \cdot t_k + \sum_{j \le m} t_j)$$

$$\phi_{snir}(k) = \Lambda \cdot (t_k \sum_{j \le m} t_j - \sum_{j \le m} t_j^2)$$

where Λ is defined as:

$$\Lambda = \frac{1}{\left(\sum_{j \le m} t_j\right)^2 - m \sum_{j \le m} t_j^2}$$

This is similar to our ψ, ϕ (see Supplemental Figure S3), except that we avoid the multiplication by Λ (because it implies division).

Snir defines the output of the site step to be the product of these matrices $[U]_{k,l}$ and $[L]_{k,l}$ by the vector Y as specified in Supplemental Figure S3. We defined the output analogously, albeit with our matrices U, L, leading to a different outcome in our site step than in Snir's. Denote Snir's outcome by $\overrightarrow{r}, \overrightarrow{s^0}$ and our outcome by $\overrightarrow{r'}, \overrightarrow{s'^0}$, then the following holds:

$$\overrightarrow{r} = \Lambda\overrightarrow{r'} \text{ and } \overrightarrow{s^0} = \Lambda\overrightarrow{s'^0}$$

Although our output of the the site step differ from Snir's, we compensate by modifying the time step accordingly, to have the combination of our site plus time step produce the exact same outcome as the combination of Snir's site plus time step. Details on our time step follow.

Snir's time step formula [Snir, 2020, Lemmas 4] calculates the predicted epigenetic age $t_{snir,j}$ for individual j as follows:

$$t_{snir,j} = \frac{\sum_{i\leq n} r_i(\hat{s}_{i,j} - s_i^0)}{\sum_{i\leq n} r_i^2}$$

Using the identities $\overrightarrow{r} = \Lambda\overrightarrow{r'}$ and $\overrightarrow{s^0} = \Lambda\overrightarrow{s'^0}$ we see that $t_{snir,j}$ is equal to our t_j that was defined to be:

$$t_j = \frac{\sum_{i\leq n} r'_i(\Lambda^{-1}\hat{s}_{i,j} - s'^0_i)}{\sum_{i\leq n}(r'_i)^2}$$

We securely calculate the numerator and denominator per individual j denoted by $t_{\mathsf{num},j}$ and $t_{\mathsf{denom},j}$ and pass them to the site step for the next iteration. Note that, at this point, our t_j and Snir's $t_{snir,j}$ are indeed equal, except that ours are represented by their pair of numerator and denominator. In the final iteration the MLE passes to CSP the ciphertexts for $t_{\mathsf{num},j}$ and t_{denom} who decrypts and divides to compute the output values t_j, which –as we have showed– is exactly the same value as Snir's.

Now we explain how to proceed to the site step of the next iteration. The updated value of Λ for the next iteration can be defined using $\overrightarrow{t_{\mathsf{num}}}$ and t_{denom} as follows:

$$\Lambda = \frac{1}{\left(\sum_{j\leq m} \frac{t_{\mathsf{num},j}}{t_{\mathsf{denom}}}\right)^2 - m\sum_{j\leq m}(\frac{t_{\mathsf{num},j}}{t_{\mathsf{denom}}})^2}$$

which, following mathematical simplifications, is equivalent to:

$$\Lambda = \frac{(t_{\mathsf{denom}})^2}{\left(\sum_{j\leq m} t_{\mathsf{num},j}\right)^2 - m\sum_{j\leq m}(t_{\mathsf{num},j})^2}$$

It is noted that in contrast to the initial definition of Λ above, the numerator is now $(t_{\mathsf{denom}})^2$ (rather than 1) which needs to be considered during the next iteration. Subsequently, we repeat the site step using the newly computed numerators and denominator for the age values calculated by the time step, multiplied by $(t_{\mathsf{denom}})^2$. Consequently, The updated values of ϕ and ψ computed in our protocol are equivalent to the following:

$$\psi(t_{\mathsf{num},k}, t_{\mathsf{denom}}) = (t_{\mathsf{denom}})^2 \cdot (-m \cdot \frac{t_{\mathsf{num},k}}{t_{\mathsf{denom}}} + \sum_{j\leq m} \frac{t_{\mathsf{num},j}}{t_{\mathsf{denom}}})$$

$$\phi(t_{\mathsf{num},k}, t_{\mathsf{denom}}) = (t_{\mathsf{denom}})^2 \cdot (\frac{t_{\mathsf{num},k}}{t_{\mathsf{denom}}} \sum_{j \leq m} \frac{t_{\mathsf{num},j}}{t_{\mathsf{denom}}} - \sum_{j \leq m} (\frac{t_{\mathsf{num},j}}{t_{\mathsf{denom}}})^2)$$

After some mathematical simplifications:

$$\psi(t_{\mathsf{num},k}, t_{\mathsf{denom}}) = (-m \cdot t_{\mathsf{num},k} + \sum_{j \leq m} t_{\mathsf{num},j}) \cdot t_{\mathsf{denom}}$$

$$\phi(t_{\mathsf{num},k}) = t_{\mathsf{num},k} \sum_{j \leq m} t_{\mathsf{num},j} - \sum_{j \leq m} t^2_{\mathsf{num},j}$$

We note that these are identical to the values of ϕ and ψ in Supplemental Figure S3. Henceforth, we can proceed to the time step calculation following the same method described above and perform additional iterations if required. □

Supplemental Note 2: Proof of Theorem 1, Privacy

We first prove that the protocol in Supplemental Figure S5 securely realizes the output revealing EPM functionality (Supplemental Figure S6). We then extend the analysis to show that the extended protocol (cf. Results) securely realizes the EPM functionality (Figure 1 in Methods).

Proof (of Theorem 1, Privacy, for the simplified functionality (Supplemental Figure S6)). Denote by $J \subseteq [m]$ the set of data owners corrupted by the adversary. We consider three cases: the adversary controls also MLE or CSP or neither (controlling both servers is disallowed in the two-server model). Privacy in the third case follows immediately from any of the preceding two cases, because the MLE and CSP have no input and the output is public; we therefore focus on the first two cases.

Case I – the adversary controls J and MLE. We construct a ppt simulator Sim_1 that receives the input of parties in J (MLE has no input), the outputted e-ages, and the leakage profile (public parameters and $\vec{t}_{\mathsf{num}}, t_{\mathsf{denom}}$), and produces a simulated-view for the adversary controlling J and MLE. The view includes the inputs of all corrupt parties, their random tape consisting of uniformly random values (of the required length) sampled by Sim_1, and a simulated view of the messages they receive throughout the protocol constructed by Sim_1 constructed as follows:

- Sim_1 honestly generates $(pk, sk) \leftarrow \mathsf{KeyGen}(1^\lambda, N)$ and adds pk to the simulated view (in place of the message received from CSP during setup).
- For every honest DO_j (i.e., $j \notin J$), Sim_1 generates n independent random encryptions of zero under pk and adds them to the view (in place of encrypted inputs received by MLE from the honest DO_j's during data upload).
- For every corrupt DO_j (i.e., $j \in J$), Sim_1 encrypts computes $\mathsf{ctxt}_j \leftarrow \mathsf{Enc}(pk, (\hat{s}_{i,j})_{i \in [n]})$ using the random coins specified in the random tape of DO_j, and adds these ciphertexts to the view (in place of encrypted inputs received by MLE from the corrupt DO_j's during data upload).
- Sim_1 adds to the view $(\vec{t}_{\mathsf{num}}, t_{\mathsf{denom}})$ (in place of the e-ages published by CSP in the output post-processing phase).

We prove that the joint distribution of the real view together with the output of the protocol is computationally indistinguishable from the joint distribution of the simulated view together with the output of the functionality. This is proven via the following hybrids.

$\mathcal{H}_0$ is the joint distribution of the adversary's view and protocol's output in a real execution of the protocol.

$\mathcal{H}_1$ is similar to $\mathcal{H}_0$, except that, in the view, the random tape, public key, and messages received during the output phase are as in the simulated view. Observe that $\mathcal{H}_0$ and $\mathcal{H}_1$ are identically distributed (because the random tape is selected uniformly at random, the keys are generated using an honest execution of KeyGen and the simulated messages for the output are the output provided to the simulator.

$\mathcal{H}_2$ is similar to $\mathcal{H}_1$ except that we replace the output of the protocol by the output of the functionality $(\vec{t}_{\mathsf{num}}, t_{\mathsf{denom}})$, and also replace the output values published by the CSP upon the termination of the output post-processing phase by these same values $(\vec{t}_{\mathsf{num}}, t_{\mathsf{denom}})$ (as used the simulated view). Observe that $\mathcal{H}_1$ and $\mathcal{H}_2$ are identically distributed because the output of the protocol and the functionality are identically distributed (by the correctness of the protocol), which in turn implies that also the output values published by the CSP upon the termination of the output post-processing phase are identically distributed to the output of the functionality (here we rely on the fact that all parties receive the same output).

$\mathcal{H}_3$ is the joint distribution of the simulated view (as computed by Sim_1) and the output of the functionality. That is, $\mathcal{H}_3$ is similar to $\mathcal{H}_2$ except that the view is the simulated view, that is, the ciphertexts received from honest DO_j's are all replaced by encryptions of zero. By the semantic security of the encryption scheme, $\mathcal{H}_2 \approx_c \mathcal{H}_3$.

We conclude that $\mathcal{H}_3 \approx_c \mathcal{H}_0$. That is, the joint distribution of the simulated view and the output of the functionality is computationally indistinguishable from the joint distribution of the view of the adversary and the output of the protocol.

Case II – the adversary controls J and CSP. We construct a ppt simulator Sim_2 that receives the public parameters, the input of parties in J (CSP has no input), the outputted e-ages and the leakage profiles, and produces a simulated-view for the adversary controlling J and CSP. The view includes the inputs of all corrupt parties, their random tape consisting of uniformly random values (of the required length) sampled by Sim_2, and a simulated view of the messages they receive throughout the protocol constructed by Sim_2 as follows.

– Sim_2 executes $(pk, sk) \leftarrow \mathsf{KeyGen}(1^\lambda, N)$ while using the randomness as set by Sim_2 in the random tape of the CSP, and adds pk to the view (in place of the public key received by the corrupt DO_j's during setup).
– Sim_2 computes $\vec{\boldsymbol{t}}_{\mathsf{num}} \leftarrow \mathsf{Enc}(pk, \vec{t}_{\mathsf{num}})$ and $\boldsymbol{t}_{\mathsf{denom}} \leftarrow \mathsf{Enc}(pk, t_{\mathsf{denom}})$, and adds the ciphertexts $(\vec{\boldsymbol{t}}_{\mathsf{num}}, \boldsymbol{t}_{\mathsf{denom}})$ to the view (in place of the ciphertexts published by the MLE upon the termination of the secure computing phase).
– Sim_2 adds to the view the cleartext values $(\vec{t}_{\mathsf{num}}, t_{\mathsf{denom}})$ (in place of the output values published by the CSP upon the termination of the output post processing phase).

We prove that the joint distribution of the real view together with the output of the protocol is computationally indistinguishable from the joint distribution of the simulated view together with the output of the functionality. This is proven via the following hybrids.

$\mathcal{H}_0$ is the joint distribution of the adversary's view and protocol's output in a real execution of the protocol.

$\mathcal{H}_1$ is similar to $\mathcal{H}_0$, except that, in the view, the random tape, public key. Observe that $\mathcal{H}_0$ and $\mathcal{H}_1$ are identically distributed (because the random tape is selected uniformly at random, the keys are generated using an honest execution of KeyGen and are consistent with the random tape of

the CSP as set by Sim_2, and the simulated messages for the output are the output provided to the simulator.

$\mathcal{H}_2$ is the joint distribution of the simulated view (as computed by Sim_2) and the output of the functionality. That is $\mathcal{H}_2$ is similar to $\mathcal{H}_1$ except that we replace the output of the protocol by the output of the functionality $(\vec{t}_{\mathsf{num}}, t_{\mathsf{denom}})$, and also: replace the cleartext values published by the CSP upon the termination of the output post-processing phase by these same values $(\vec{t}_{\mathsf{num}}, t_{\mathsf{denom}})$, and replace the ciphertexts published by the MLE upon the termination of the secure computing phase by the encryption of these values using pk, i.e., by $(\vec{\boldsymbol{t}}_{\mathsf{num}}, \boldsymbol{t}_{\mathsf{denom}})$ defined by $\vec{\boldsymbol{t}}_{\mathsf{num}} \leftarrow \mathsf{Enc}(pk, \vec{t}_{\mathsf{num}})$ and $\boldsymbol{t}_{\mathsf{denom}} \leftarrow \mathsf{Enc}(pk, t_{\mathsf{denom}})$. Observe that $\mathcal{H}_1$ and $\mathcal{H}_2$ are identically distributed because the output of the protocol and the functionality are identically distributed (by the correctness of the protocol), which in turn implies that also the output values published by the CSP upon the termination of the output post-processing phase are identically distributed to the output of the functionality (here we rely on the fact that all parties receive the same output).

We conclude that $\mathcal{H}_2 \approx_c \mathcal{H}_0$. That is, that the joint distribution of the simulated view and the output of the functionality is computationally indistinguishable from the joint distribution of the view of the adversary and the output of the protocol. □

Proof (of Theorem 1, Privacy). We next sketch how we extend the above proof to show that our full protocol (cf. Results) securely realizes the EPM functionality (Figure 1 in Methods).

We first analyze the extension of the protocol for hiding the outputted numerators. The proof of privacy extends for the case of hiding $\overrightarrow{t_{\mathsf{num}}}$ (see Results section) by letting the simulator generate uniformly random values in place of the masked values for the honest data owners – resulting in an identical distribution (since the adversary has no information of what mask is generated by honest data owners). The formal proof is via extending the hybrid argument accordingly.

We next analyze the protocol extension for hiding both the numerators and the denominator. Extending the proof of privacy for the case of hiding also t_{denom} is done by considering two cases: either the adversary controls MLE or CSP (recall that it cannot control both). In case the adversary controls MLE (and any number of the data owners), the simulator generates a fresh encryption of zero in place of the ciphertext $\boldsymbol{t'}^{-1}_{\mathsf{denom}}$ received by MLE from the CSP–relying on the semantic security of the FHE scheme–, and uses the output values t_j to simulate the messages received by the corrupt data owners DO_j in the output post processing phase (recall that the simulator is provided the input and output of corrupt parties). In case the adversary controls CSP (and any number of the data owners), the simulator uses a uniformly random value in place of the t'_{denom} computed by MLE in a real execution of the protocol – resulting in identical distributions.

Supplemental Note 3: Proof of Theorem 2, Complexity

Proof (of Theorem 2, Size). We analyze the number of homomorphic operations computed by the MLE in our privacy preserving EPM protocol. Homomorphically computing ψ_k takes $m+1$ homomomorphic additions and at most 2 homomomorphic multiplications. Homomorphically computing U will therefore take $O(m \cdot (m+3)) = O(m^2 + 3m)$ homomomorphic multiplications and addition operations. Homomorphically computing ϕ_k takes $2m$ homomomorphic additions, 1 homomomorphic subtractraction, and 1 homomomorphic multiplication. So, homomorphically computing L will take $O(m \cdot (2m+2) = O(2m^2 + 2m)$ homomomorphic multiplications and addition operations. Homomorphically computing $\vec{r'} = U \cdot Y$ and $\overrightarrow{s'^{0}} = L \cdot Y$ is done as follows to

utilize the sparsity of U and L: Multiplying each row of U or L by Y requires computing only m homomorphic multiplication and addition operations (because the row has only m non-zero entries). So, doing so for all n row takes nm homomorphic operations. Specifically, computing $\overrightarrow{r'}$ will take in total $O(m^2 + 3m + 2mn) = O(m^2 + m(3 + 2n))$ and computing $\overrightarrow{s'^0}$ will take $O(2m^2+2m+2mn) = O(2m^2+2m(1+n))$. A summary of the site step total time complexity will be: $O(3m^2+m(5+4n))$. Computing Λ^{-1} takes $O(2m+1)$ homomorphic additions and multiplications. Computing t_{num} takes $O(n)$ homomorphic additions and multiplications. Computing t_{denom} takes $O(n)$ homomorphic additions and multiplications. A summary of the time step total complexity will be: $O(2n + 2m + 1)$ homomorphic operations. Adding the complexity of both steps results in the following complexity for a single iteration of the algorithm: $O(3m^2 + m(7 + 4n) + 2n + 1)$. □

Proof (of Theorem 2, Depth). We analyze the multiplicative-depth of the circuit homomorphically evaluated by the MLE in our privacy preserving EPM protocol. For each variable x in our protocol, we denote the $\times$-depth of computing its value in iteration k by $\mathsf{d}(x;k)$. We show by induction that in each iteration k $\mathsf{d}(\overrightarrow{t_{\mathsf{num}}};k) = \mathsf{d}(\overrightarrow{t_{\mathsf{denom}}};k) = 3(k-1)$. The base case is the first iteration. In this iteration, $\overrightarrow{t}$ consists of random values generated by the MLE and processed in cleartext. So, $\mathsf{d}(\overrightarrow{\phi};1) = \mathsf{d}(\overrightarrow{\psi};1) = 0$, implying that $\mathsf{d}(\overrightarrow{r'};1) = \mathsf{d}(\overrightarrow{s'^0};1) = 0$ (where we assume that the underlying FHE supports plaintext vs. ciphertext multiplication being processed using homomorphic additions only as to incur no added $\times$-depth), and also $\mathsf{d}(\Lambda^{-1};1) = 0$; so, $\mathsf{d}(t_{\mathsf{num}};1) = \mathsf{d}(t_{\mathsf{denom}};1) = 0$.

Consider next the kth iteration for $k > 1$. The variables $\overrightarrow{t_{\mathsf{num}}}$ and t_{denom} entering this iteration are in encrypted form. Denote $\mathsf{d}(\overrightarrow{t_{\mathsf{num}}};k-1) = a$ and $\mathsf{d}(t_{\mathsf{denom}};k-1) = b$. So,

$$\mathsf{d}(\psi;k) = \max\{\mathsf{d}(\overrightarrow{t_{\mathsf{num}}};k-1), \mathsf{d}(t_{\mathsf{denom}};k-1)\} + 1 = \max\{a,b\} + 1$$

and

$$\mathsf{d}(\phi;k) = \mathsf{d}(t_{\mathsf{num}};k-1) + 1 = a + 1$$

implying that

$$\mathsf{d}(\overrightarrow{r'};k) = \mathsf{d}(\psi;k) + 1 = (\max\{a,b\} + 1) + 1 = \max\{a,b\} + 2$$

and

$$\mathsf{d}(\overrightarrow{s'^0};k) = \mathsf{d}(\phi;k) + 1 = (a+1) + 1 = a + 2.$$

Likewise,

$$\mathsf{d}(\Lambda^{-1};k) = \mathsf{d}(\overrightarrow{t_{\mathsf{num}}};k-1) + 1 = a + 1.$$

So,

$$\mathsf{d}(\overrightarrow{t_{\mathsf{num}}};k) = 1 + \max\{\mathsf{d}(\overrightarrow{r'};k), \mathsf{d}(\Lambda^{-1};k) + 1\} = 1 + \max\{\max\{a,b\} + 2, (a+1) + 1\} = \max\{a,b\} + 3$$

and

$$\mathsf{d}(t_{\mathsf{denom}};k) = \mathsf{d}(\overrightarrow{r'};k) + 1 = (\max\{a,b\} + 2) + 1 = \max\{a,b\} + 3.$$

By the induction hypothesis $a = b = 3(k-1)$. Assigning these value for a, b, we get that $\mathsf{d}(\overrightarrow{t_{\mathsf{num}}};k)$ and $\mathsf{d}(t_{\mathsf{denom}};k)$ are equal to $\max\{a,b\} + 3 = 3(k-1) + 3 = 3k$, which proves the desired. □

Supplemental Note 4: Proof of Maximum Plaintext Modulus Value (Supplemental Figure S4)

Claim. Let $m, n, \mathsf{iter}, \ell$ be the EPM functionality parameters. Let σ, τ, N_0 be as set in Figure S5. In any executing of the protocol in Figure S5 on these EPM parameters, any methylation values in $(0, 1)$ and e-ages initialization range $[0, \tau]$, all plaintext values are strictly smaller than N_0.

Proof. In high level, the algorithm in Figure S4 computes a recurrence on the value of t_{num} in the final iteration of the protocol in Figure S5, when executed on maximal methylation and age values (σ and τ). This serves as an upper bound to all other values computed during our protocol, because the value of t_{num} in the final iteration is an upper-bound on all other values computed throughout the protocol execution. Moreover, its value is maximal when the methylation and age values are equal to σ and τ respectively.

Details on the recurrence formula and on showing that $t_{\mathsf{num}} \geq t_{\mathsf{denom}}$ follow. We proceed to find the maximum possible values: $\mathsf{max}(\overrightarrow{t_{\mathsf{num}}})$ and $\mathsf{max}(t_{\mathsf{denom}})$ over all valid inputs to the protocol during all iterations. In all calculations, we must take into account that the age values represent the epigenetic state, and therefore may also be negative.

Recall that τ is the upper bound for the age values and σ as the upper bound for the input methylation values.

We denote $(\text{var}; k)$ as the value of the variable var in iteration k.

- $\mathsf{max}(\overrightarrow{t_{\mathsf{num}}}; \mathsf{iter})$: We first define an upper bound for the maximum value of r'_i and $\left(\Lambda^{-1}\hat{s}_{i,j} - {s'}^0_i\right)$ appearing in the formula of t_{num} specified in Supplemental Figure S3.
  - We begin by defining the maximal value in the vector $\overrightarrow{r'}$.
    By inspecting the formula, we conclude that the maximum value can be calculated as:
    $$m \cdot (2m(\tau; \mathsf{iter})) \cdot \sigma \cdot (t_{\mathsf{denom}}; \mathsf{iter} - 1)$$
  - Next, we wish to maximize $\Lambda^{-1}\hat{s}_{i,j}$.
    We find the maximum value here to be:
    $$(m(\tau; \mathsf{iter}))^2 \cdot \sigma$$
  - Due to the negative sign before ${s'}^0_i$, we wish to express the minimal possible value in the vector $\overrightarrow{s'^0}$. We define this value as:
    $$m \cdot (-2m \cdot (\tau; \mathsf{iter})^2) \cdot \sigma$$
  - Concluding from the above, we can define that:
    $$\begin{aligned}\mathsf{max}(\overrightarrow{t_{\mathsf{num}}}; \mathsf{iter}) &= n \cdot 2m^2 \cdot (\tau; \mathsf{iter}) \cdot (t_{\mathsf{denom}}; \mathsf{iter} - 1) \cdot \sigma \cdot (m^2(\tau; \mathsf{iter})^2\sigma + (2m^2(\tau; \mathsf{iter})^2) \cdot \sigma) = \\ &= n \cdot 6m^4(\tau; \mathsf{iter})^3\sigma^2 \cdot (t_{\mathsf{denom}}; \mathsf{iter} - 1)\end{aligned}$$
- $\mathsf{max}(t_{\mathsf{denom}}; \mathsf{iter})$: As depicted in Supplemental Figure S3, we wish to maximize the value $\sum_{i \leq n} {r'}^2_i$. In order to achieve this, we can use the definition of $\mathsf{max}(\overrightarrow{r'})$ from the previous step. We then conclude that:
  $$\mathsf{max}(t_{\mathsf{denom}}; \mathsf{iter}) = (\mathsf{max}(\overrightarrow{r'}))^2 = n \cdot 4m^4 \cdot (\tau; \mathsf{iter})^2\sigma^2(t_{\mathsf{denom}}; \mathsf{iter} - 1)^2$$

We now need to determine which of the values is larger. In the first iteration, we define $(t_{\mathsf{denom}}; \mathsf{iter}-1) = 1$, therefore, for this step it is clear that the value of $\max(\overrightarrow{t_{\mathsf{num}}})$ is the larger one as:

$$n \cdot 6m^4 \cdot (\tau; \mathsf{iter})^3\sigma^2 \cdot 1 > n \cdot 4m^4 \cdot (\tau; \mathsf{iter})^2\sigma^2 \cdot 1$$

For the next iteration, we set $(\tau; \mathsf{iter}) = \mathsf{max}(\overrightarrow{t_{\mathsf{num}}}; \mathsf{iter}-1)$ as this is now the largest age value. We then proceed to evaluate the values of $\mathsf{max}(\overrightarrow{t_{\mathsf{num}}}; \mathsf{iter})$ and $\mathsf{max}(t_{\mathsf{denom}}; \mathsf{iter})$ as follows:

$$\mathsf{max}(\overrightarrow{t_{\mathsf{num}}}; \mathsf{iter}) = n \cdot 6m^4 \cdot (\tau; \mathsf{iter})^3\sigma^2 \cdot (t_{\mathsf{denom}}; \mathsf{iter}-1)$$

$$\mathsf{max}(t_{\mathsf{denom}}; \mathsf{iter}) = n \cdot 4m^4 \cdot (\tau; \mathsf{iter})^2\sigma^2 \cdot (t_{\mathsf{denom}}; \mathsf{iter}-1)^2$$

We now need to determine whether $3 \cdot (\tau; \mathsf{iter}) \geq 2 \cdot (t_{\mathsf{denom}}; \mathsf{iter}-1)$.

The above can be written as:

$$3 \cdot (n \cdot 6m^4 \cdot (\tau; \mathsf{iter}-1)^3\sigma^2) \geq 2 \cdot (n \cdot 4m^4 \cdot (\tau; \mathsf{iter}-1)^2\sigma^2)$$

From the above, it is clear that $9 \cdot (\tau; \mathsf{iter}-1) > 4$.

This implies that $\mathsf{max}(\overrightarrow{t_{\mathsf{num}}; \mathsf{iter}}) > \mathsf{max}(t_{\mathsf{denom}}; \mathsf{iter})$.

In Supplemental Figure S4, the value of N_0 is derived from the above formula of $\mathsf{max}(\overrightarrow{t_{\mathsf{num}}}; \mathsf{iter})$.

As τ and σ are upper bounds on age and methylation values respectfully and N_0 is multiplied by a value of 2 in the final stage of the algorithm, we conclude that the N_0 outputted from the algorithm is larger than all values computed during our secure protocol. □

Supplemental Note 5: EPM Quantitative Insights

To offer insights into the number of individuals required for achieving a high level of accuracy for the EPM algorithm, we present our simulated estimation. Our results indicate that an amount of over 5050 individuals should be sufficient to fulfill the high accuracy requirement.

Due to the lack of available methylation datasets with large quantities of individuals, we generated a synthetic dataset and conducted a simulation. To generate our synthetic data, we used rates and methylation at birth values in ranges extracted from our empirical evaluation which is based on real human data. Next we generated random chronological age values between 0 and 99 and calculated methylation values per individual per site. We then ran the EPM site step on the above methylation values and rates to calculate epigenetic ages which will serve as our ground truth reference.

Using our generated chronological ages and calculated methylation values as input, we ran the EPM on subsets of our dataset containing growing amounts of individuals. For each subset we measured the difference of the epigenetic ages calculated for the first 50 individuals by the EPM to our ground truth epigenetic ages. Our data generation and simulation are detailed in Supplemental Figure S7.

We repeated this simulation for datasets containing 7,000, 10,000 and 20,000 individuals. Each dataset was generated and tested twice to introduce additional data randomness. We defined our point of saturation as $RSS < 1$. This indicates that in average, each predicted epigenetic age has a less than $\frac{1}{50}$ square difference in years to its matching ground truth value. Supplemental Figure S8 depicts the results from a dataset of 10,000 individuals, where the point of saturation is reached at 5050 individuals. Our other datasets showed similar behaviour.

Our experiments lead us to a conclusion that a dataset containing over 5000 individuals should provide a high level of accuracy for the EPM algorithm.

References

Sagi Snir. Epigenetic pacemaker: closed form algebraic solutions. *BMC genomics*, 21(2):1–11, 2020.
